# Pathways of Photocatalytic Oxidation of Formic Acid on Dry and Hydrated Anatase TiO_2_ Surfaces

**DOI:** 10.1021/acscatal.5c01848

**Published:** 2025-06-18

**Authors:** Chiara Daldossi, Cristiana Di Valentin, Annabella Selloni

**Affiliations:** † Department of Materials Science, 189823 University of Milano-Bicocca, via R. Cozzi 55, Milano 20125, Italy; ‡ Department of Chemistry, 6740 Princeton University, Princeton, New Jersey 08544, United States

**Keywords:** photocatalysis, formic acid, TiO_2_, H_2_O, hybrid density functional theory, Heyd–Scuseria–Ernzerhof HSE06

## Abstract

The photocatalytic oxidation of formic acid (FA), which is one of the most abundant volatile organic compounds, is a promising air remediation technology inspired by nature. However, the detailed mechanism of this photocatalytic reaction on the surface of TiO_2_, a typical photocatalyst, is not yet well-understood. In this work, we present a computational mechanistic study of the thermal vs photocatalytic oxidation of FA on dry and hydrated anatase TiO_2_ (101) surfaces, based on periodic hybrid density functional theory (DFT) calculations, in which the photo-oxidation is treated as an excited-state process in a constrained triplet spin state. We first compare the adsorption modes of FA on the anatase (101) surface in the ground and excited states, followed by identification of the corresponding reaction intermediates that lead to the formation of CO_2_. We unveil the pivotal role of photogenerated holes localized at surface under-coordinated oxygen sites in mediating the C–H bond cleavage, thereby promoting CO_2_ formation through a highly stable intermediate and an exergonic reaction step. Further investigation of the effect of coadsorbed water molecules shows that hydrogen bonding with water stabilizes FA in a monodentate configuration. This is favored over the unreactive bidentate structure that is the most stable under dry conditions, thus providing insight into the experimentally observed increase of the reaction rate in the presence of water.

## Graphical abstract

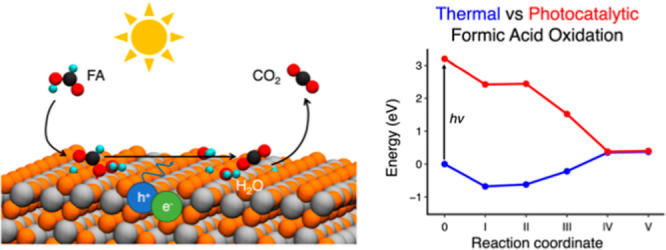

## 1 Introduction

Volatile organic compounds (VOCs), typically defined as the organic compounds with the boiling point in the range 50 to 260 °C at standard atmospheric pressure, are major air pollutants and pose a serious threat to human health and the eco-environment due to their properties of volatility, toxicity, and diffusivity. Among the most common approaches to VOCs elimination,^1–6^ photocatalytic oxidation (PCO) is a promising air remediation technology that can oxidize low concentrations of VOCs under ambient conditions with high economic feasibility and low levels of secondary pollutant generation. PCO is a gas–solid heterogeneous catalytic reaction driven by light at room temperature, where a semiconductor photocatalyst is used in the presence of a light source to degrade pollutants into more stable (typically oxidized) products, such as CO_2_ and H_2_O.^7–9^

The photocatalytic oxidation of VOCs adsorbed on the surfaces of TiO_2_, one of the most widely used photocatalysts, provides a potential cost-effective method for applications in air purification and self-cleaning windows.^10^ Among the different polymorphs of TiO_2_, the anatase phase, with nanoparticles primarily exposing (101) facets,^11–14^ is considered the most active in photocatalysis.^15,16^ Equilibrium-shaped anatase nanoparticles are truncated bipyramids typically dominated by the (101) facet.^17,18^ Even though the (001) surface is known to be more reactive, its limited abundance under typical synthesis and operating conditions makes the (101) surface a more representative choice for mechanistic investigations since it is expected to be the main site where surface reactions, including VOCs oxidation, occur. The surface energies of low-index anatase facets are in the order (110) > (001) > (010) > (101).^11,19^ The high specific surface energy and high reactivity of the (001) facet compared to that of (101) are the reason for its quick disappearance during the crystal growth process.^20^ Anatase TiO_2_ nanoparticles used in photocatalytic applications typically deviate from the ideal bulk crystal structure as they expose a variety of defect-rich sites that can modify the surface structural, electronic, and chemical properties. These include structural irregularities such as steps and edges, which feature undercoordinated atoms that significantly enhance the surface reactivity. In addition, intrinsic defects, such as oxygen vacancies, interstitial atoms, or dopants, introduce localized electronic states and alter the charge distribution, further influencing the photocatalytic behavior. These defect sites can serve as active centers for adsorption, charge trapping, and reaction initiation. Under realistic ambient conditions, the adsorption of environmental species, including water and oxygen,^21^ also plays a crucial role in tuning the surface reactivity and the overall photocatalytic performance of the material.^14^ In the present work, we chose to model the stoichiometric anatase (101) surface, which is the thermodynamically most stable and most exposed facet in equilibrium-shaped anatase nanoparticles. Moreover, by focusing on the ideal (defect-free) surface, we can isolate fundamental aspects of the VOCs’ adsorption and reaction mechanism.

Photocatalytic reactions on TiO_2_ are initiated by ultraviolet light that excites electrons from the valence to the conduction band of the semiconductor, generating electron–hole (e^–^–h^+^) pairs. These are photogenerated in the bulk of the semiconductor, but electrons and holes that do not recombine tend to migrate to the surface, where they can participate in redox reactions. In particular, the holes that successfully reach the surface can oxidize the organic pollutants adsorbed on the TiO_2_ surface to form CO_2_.^22^ To study such a photocatalytic reaction, here, we use a simplified approach based on spin-constrained density functional theory (DFT), where the spin of the system is locked in the lowest triplet state to mimic the excited state, with an electron in the conduction band and a hole in the valence band.^14,15,23–25^ In this approach, the ground and photoexcited states can be correctly calculated by DFT because they are the lowest energy states of singlet and triplet spin multiplicity, respectively.^14,15^

Formic acid (FA) is one of the most abundant VOCs^10^ and the simplest carboxylic acid. Experimentally, evidence of both molecular and dissociative adsorption has been reported for FA on dry anatase (101).^26–31^ In particular, under dry conditions, dissociative adsorption to form bridging bidentate formate is prevalent,^29,32–34^ but smaller quantities of physisorbed and chemisorbed molecular formic acid are also observed.^28,31^ On the theoretical side, adsorption of formic acid on anatase (101) has been widely investigated by density functional theory (DFT), and different models have been proposed depending on the exchange–correlation functional used. Corrections for dispersion forces were reported to be important to describe the relative stability of the adsorption structures with the standard gradient-corrected PBE^35^ density functional.^36^ After adsorption on the TiO_2_ surface, formic acid and formate species are photocatalytically oxidized to CO_2_ and H_2_O in the presence of O_2_.^9,37–41^ Given the coexistence of protonated and unprotonated FA on the TiO_2_ surface, the formation of CO_2_ could result from the decomposition of both species. Fourier transform infrared (FTIR) spectroscopy experiments of Liao et al.^28^ concluded that molecularly adsorbed formic acid is the most important species for CO_2_ production upon UV irradiation as this is responsible for the initial production of a high concentration of CO_2_ followed by slower photodecomposition of formate at longer illumination times.

Given the ubiquitous presence of water under ambient conditions, it is also important to consider the effect of water during photocatalytic reactions of the adsorbed molecules on TiO_2_. On the hydrated anatase TiO_2_ (101) surface, changes in the Fourier transform infrared spectra compared to those obtained on dry surfaces indicate the presence of interaction between adsorbed formate and water molecules.^28,31^ An early computational study by Vittadini et al.,^42^ based on the GGA–PBE density functional,^35^ found that molecular monodentate adsorption is the most stable for FA on the clean surface, while on the hydrated surface, the dissociated monodentate configuration, with the acidic proton transferred to a surface bridging oxygen atom (O_2c_), is preferred due to the formation of a hydrogen bond between a formate oxygen and the nearby water molecule.^43^ Many experimental studies conducted in the presence of water also show an increase in the photodegradation rates of both formic acid and formate species.^28,31,32^ Although the mechanism for the water enhancement effect is still debated, water coverage has been found to play a crucial role in determining the photocatalytic reaction rate. Liao et al.^40^ reported that 60% saturation coverage of water molecules was required to substantially increase the formate photooxidation on TiO_2_, while Miller et al.^32^ observed that only physisorbed water and not strongly adsorbed water molecules enhance the formic acid photocatalytic degradation rate. Miller et al.^32^ also suggested that the addition of water displaces the weakly adsorbed formic acid, making its adsorption on the TiO_2_ surface more difficult, while it also increases formic acid decomposition, causing the conversion of bidentate formate to monodentate formate that is more reactive.^32,33,40,44,45^ On the other hand, spectroscopy experiments by Mattsson and Osterlund^41^ suggested that formate ions are present on anatase and the water on the surface displaces the adsorbed organic acids and impedes the interfacial charge transfer,^41^ thus reducing the decomposition rate.

In this work, we aim to elucidate the mechanism of the photocatalytic oxidation of formic acid on TiO_2_ and identify the effect of water, which is experimentally known to improve the reaction rate. When the reaction is activated by light, the overall PCO reaction relies on three critical steps: (1) VOC adsorption on the catalyst surface; (2) absorption of light and generation of charge carriers; and (3) reactions between the adsorbed VOCs and the charge carriers, leading to the formation and subsequent desorption of photo-oxidation products. Thus, understanding the surface chemistry is key to unraveling the PCO process. Given that the relative stability of molecular and dissociated formic acid adsorption modes predicted by computational studies depends significantly on the method used, here, we apply hybrid density functional methods, both because they show better agreement with experimental data^42,46–48^ and because, by incorporating a portion of exact (Hartree–Fock type) exchange and reducing the self-interaction error inherent in local and semilocal DFT approaches, they provide a more accurate representation of the TiO_2_ band gap. This improved accuracy is crucial for a reliable description of photocatalytic processes. Through hybrid DFT calculations, we first study the reaction in the dark and under light illumination on the dry anatase TiO_2_ (101) surface by determining the formic acid adsorption modes and optimizing the reaction intermediates leading to CO_2_ formation in both the ground (singlet) and photoexcited (triplet) states. Next, we extend our study to the hydrated TiO_2_ surface to investigate the effect of coadsorbed water molecules.

## 2 Computational Methods

All density functional theory (DFT) calculations were performed with the CRYSTAL17 ^49^ package, where the Kohn–Sham orbitals are expanded in Gaussian-type orbitals. The all-electron basis sets employed were Ti 86-411­(d41) and O 8-411­(d1) for the atoms of TiO_2_ and H 511­(p1), C 6-311­(d11), and O 8-411­(d11) for the atoms of formic acid and water.

To accurately describe the electronic structure of anatase TiO_2_, the range-separated Heyd–Scuseria–Ernzerhof HSE06 hybrid functional^50^ was used for all calculations. The HSE hybrid functional consistently predicts more accurate structural and electronic properties, including the band gap, than standard local and semilocal density functional approaches such as the local density approximation (LDA) or the generalized gradient approximation (GGA). Even though it is known that hybrid functionals incorporating approximately 20–25% exact Hartree–Fock exchange tend to overestimate the band gap of anatase, the original parametrization of the standard HSE06 functional (i.e., HSE with mixing α = 0.25 and screening μ = 0.20), well satisfies the general criteria for accurate electronic structure calculations.^50–55^ Using the standard HSE06 functional, we obtain a band gap of 3.65 eV for bulk anatase, in good agreement with previously reported values (ranging from 3.58 to 3.89 eV)^53,54,56^ and experiments.^14^

Grimme’s dispersion corrections D3 ^57^ have been included (HSE06-D3) in the calculations of intra-pair reaction intermediates to assess the effect of long-range van der Waals interactions with respect to pure HSE06 calculations.

Cutoff limits in the evaluation of Coulomb and exchange series/sums appearing in the self-consistent field (SCF) equation were set to 10^–7^ for Coulomb overlap tolerance, Coulomb penetration tolerance, exchange overlap tolerance, and exchange pseudo-overlap in the direct space and 10^–14^ for exchange pseudo-overlap in the reciprocal space. The condition for the SCF convergence was set to 10^–6^ hartree on the total energy difference between two subsequent cycles.

To model the anatase TiO_2_ (101) surface, we used a periodically repeated slab of 4 layers with a 1 × 3 surface supercell, with a total of 144 atoms. On the perfect anatase (101) surface, rows of fully coordinated 6-fold (Ti_6c_) and undercoordinated 5-fold (Ti_5c_) Ti atoms along the [010] are connected by 2-fold undercoordinated (O_2c_) and 3-fold fully coordinated O_3c_ atoms. The system was treated as periodic along the [101] and [010] directions, while no periodic boundary conditions were imposed in the direction perpendicular to the surface. Calculations were performed by sampling the Γ point only in the first Brillouin zone. Convergence in the geometry optimization process is tested on the root-mean-square (rms) and the absolute value of the largest component of both the gradients and nuclear displacements. The default thresholds for geometry optimization within the CRYSTAL code have been used for all atoms: maximum and rms forces have been set to 4.50 × 10^–4^ and 3.0 × 10^–4^ au, respectively, and maximum and rms atomic displacements have been set to 1.80 × 10^–3^ and 1.20 × 10^–3^ au, respectively.

In all calculations, formic acid was adsorbed only on the upper surface of the slab, while the Ti and O atoms in the bottom layer were kept fixed in their bulk positions during the geometry optimization. The adsorption energies of the various intermediates or products along the reaction path in anhydrous conditions were computed according to the following equation

1 $$E_{ads}=E_{tot}-\left(E_{slab}+E_{FA}\right)$$

where *E*_tot_ is the total energy of the intermediate, *E*_slab_ is the energy of the pristine anatase (101) slab, and *E*_FA_ is the energy of isolated gas phase formic acid.

For formic acid oxidation, the vibrational frequencies of the isolated molecules and the intermediates bound to the surface were calculated under dry and hydrated conditions by using the harmonic approximation with the CRYSTAL17 code. Numerical Hessian matrices were obtained by finite displacements, applying ±0.003 Å shifts along the three Cartesian directions to each atom from its equilibrium position. Force components for each displacement were then used to construct the Hessian matrix, which was subsequently diagonalized to obtain the vibrational frequencies corresponding to each mode.

Using the calculated frequencies, we included the zero-point energy (ZPE) corrections and entropic contributions in the revised energy profiles. Enthalpic and entropic contributions were computed under standard-state conditions by applying the ideal gas, rigid rotor, and harmonic oscillator approximations to account for the translational, rotational, and vibrational terms of the isolated formic acid and CO_2_, along with only vibrational terms for the surface-bound intermediates. Based on these, we could derive total Gibbs free energies (*G*_tot_) as

2 G=EL+ZPE+ET+PV−TS

where EL is the electronic energy from geometry optimization calculations, ZPE is the zero-point energy, and ET is the thermal contribution to the vibrational energy at temperature *T* = 298.15 K. Next, we also derived Gibbs free energies of adsorption, in line with eq 1 of the manuscript

3 $$G_{ads}=G_{tot}-\left(G_{slab}+G_{FA}\right)$$

where *G*_tot_ is the total Gibbs free energy of the intermediate, *G*_slab_ is the Gibbs free energy of the pristine anatase (101) slab, and *G*_FA_ is the Gibbs free energy of isolated gas phase formic acid.

We did not determine the transition states and energy barriers along the investigated reaction paths because the transition state search based on the saddle point localization that is implemented in the CRYSTAL code was found to be computationally too complex and demanding in the presence of open shell systems on a periodic surface model, as considered in our study.

### 2.1 Hydrated Anatase (101) Surface

To determine the effect of coadsorbed water on the formic acid adsorption and oxidation mechanism, the introduction of explicit water molecules is necessary.^58^ Here, we restrict it to the simplest model, in which only one water molecule is added on the anatase surface slab. This approach is not intended to replicate a fully solvated interface or a bulk aqueous environment but rather aims to capture the first-order effects of hydrogen bonding interactions on the stabilization of the adsorbed intermediates. Although it is a simplification, the use of a minimal water layer has been previously employed in surface science studies and has proven to be a reliable and insightful model for understanding the initial stages of hydration and its influence on adsorption and reaction mechanisms at oxide surfaces.^45,59,60^ By employing a single water molecule, we can assess how hydrogen bond formation alters the local electronic structure, surface relaxation, and charge redistribution during formic acid adsorption and the subsequent reaction steps. Importantly, this model enables us to retain computational feasibility while performing hybrid DFT calculations, which is crucial for an accurate description of the charge localization and the electronic structure of the system.

The water molecule is molecularly adsorbed onto a surface Ti_5c_ site, with its protons pointing toward two intra-pair O_2c_ surface sites, which is the most stable configuration on the anatase TiO_2_ (101) surface according to prior work.^61^

The reaction intermediates of the formic acid oxidation in the presence of a coadsorbed water molecule were constructed based on previous studies.^32,40–42,45,59,62^ In all calculations, the formic acid and water molecules were adsorbed only on the upper surface of the slab, while the Ti and O atoms in the bottom layer were kept fixed in their bulk positions during geometry optimization.

The adsorption energies of the various intermediates or products along the reaction path on the hydrated TiO_2_ were computed according to eq 1, where *E*_tot_ is the total energy of the intermediate, *E*_slab_ is the energy of the anatase (101) surface with adsorbed undissociated H_2_O, and *E*_FA_ is the energy of isolated gas phase formic acid.

### 2.2 Lowest Excited Triplet State of the Anatase (101) Surface

Excitation of TiO_2_ by UV light absorption leads to the formation of electrons and holes that can subsequently recombine, become trapped, or migrate from the bulk of the material to the surface, where they can undergo charge transfer reactions to adsorbates. To describe the photogenerated electron–hole pairs, we performed spin-constrained TiO_2_ periodic DFT calculations using the CRYSTAL17 code. The photogenerated electron–hole pair was obtained by constraining the solution into a triplet state to mimic the excited state with a photoexcited electron in the conduction band and a hole in the valence band.^24,25^ Triplet excited state calculations are started from the singlet ground state geometry, and, through structural relaxation, the electron and hole in the triplet spin configuration become localized.^14,15^ To describe the self-trapping, the structures in the excited triplet state are computed using the HSE06 hybrid functional, which reduces the self-interaction error and delocalization of holes and electrons given by standard (semi)­local DFT. In our calculations, the localization of the photogenerated hole at either O_2c_ or O_3c_ sites was controlled by starting the geometry optimization with longer Ti–O bonds around the chosen polaron site.

In a previous study of photoexcited anatase TiO_2_ employing the B3LYP hybrid functional,^15^ spin-constrained triplet state calculations found that the photoexcited electron became localized at a Ti_6c_ subsurface site not directly connected to the surface bridging oxygen, while the hole was localized at a bridging oxygen, causing the Ti–O_2c_–Ti bonds to elongate. Differently from what is observed with the B3LYP functional,^15^ the present spin-constrained triplet state calculations employing the hybrid HSE06 functional show that the photoexcited electron in the conduction band is delocalized over several Ti_6c_ subsurface sites, while the hole is localized at one bridging oxygen. This confirms that B3LYP tends to overestimate the electron localization in TiO_2_, as discussed in ref 14. The use of the HSE06 functional, known to provide a more accurate band gap and electronic structure, thus enables a more reliable representation of the photoexcited charge distribution and is well-suited to describing the reactivity of the triplet state.^63^

When formic acid and water molecules are present on the surface, the localization of the photogenerated charges is determined by a combination of factors, including the ground state electronic structure, the states near the valence band maximum, and the ability of certain adsorbed species to stabilize the excess electron or hole via structural relaxation (polaronic effects) or charge transfer mechanisms. The localization of electrons and holes thus arises from the balance between these factors. Our calculations capture the result of these effects by allowing the electronic and atomic structure to relax in the triplet excited state used to simulate the photoexcitation.

We also recall that the wave functions obtained from unrestricted calculations are no longer eigenfunctions of the total spin operator **S** ^2^ and are thus affected by spin contamination, meaning they contain contributions from states with higher spin multiplicity. This leads to an increase in the ⟨**S** ^2^⟩ expectation value above its theoretical value for a pure spin state. It is widely agreed that, in comparison to wave function-based unrestricted Hartree–Fock (UHF) methods, spin-polarized DFT, as used in the present work, yields energies that are less affected by spin contamination.^64,65^ However, this conclusion applies mostly to systems in the ground electronic state and might be less effective for systems in their excited state.

For the adsorption energy of excited-state structures, we used eq 1, where *E*_tot_ is the total energy of the intermediate, *E*_slab_ refers to the anatase (101) surface in its excited triplet state, and *E*_FA_ is the energy of isolated gas phase formic acid.

### 2.3 Nomenclature for Formic Acid Adsorption Geometries

Adsorption of formic acid on the TiO_2_ surface can take place in a monodentate (MD) or bidentate (BD) fashion depending on the number of oxygen atoms used by the molecule to bind to the surface Ti_5c_ sites. Indeed, the carboxylic group of FA is particularly well-suited for the functionalization of TiO_2_,^21^ though other oxygen-containing functional groups, such as silanols and catechol, can be used as anchoring groups.^66^ The formic acid adsorption modes are represented schematically in Scheme 1.

**Scheme 1:**
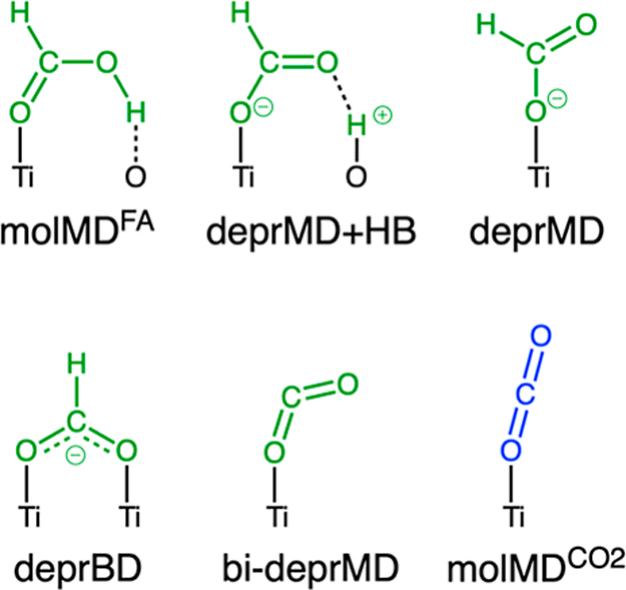
Adsorption Configurations of Formic Acid (Green) and CO_2_ (Blue) on TiO_2_ (Black)^a^

a Molecular (mol), deprotonated (depr) and bideprotonated (bi-depr) monodentate (MD), and deprotonated bidentate (BD) structures are shown. Hydrogen bonds are represented by dashed black lines.

For molecular formic acid, monodentate adsorption through the carbonyl group (denoted molMD^FA^ in Scheme 1) was found to be more stable than using the O atom of the hydroxyl group or having both oxygen atoms coordinated to the anatase surface in a bidentate mode.^42^ This monodentate adsorption mode allows the hydroxyl to form a hydrogen bond with a surface bridging oxygen. Similarly to formic acid, its oxidation product (CO_2_) also adsorbs in a molecular monodentate mode (molMD^CO_2_^ in Scheme 1)^67–69^ on the anatase (101) surface. When formic acid is deprotonated, it can bind in a monodentate fashion, with (deprMD + HB) or without formation of an H-bond (deprMD) with the dissociated proton bound to a surface O_2c_ site. Instead, in the deprotonated bidentate (deprBD) adsorption mode, the O atoms of the formate bind to two Ti_5c_ sites, while the proton binds to a surface O_2c_. Finally, the bideprotonated formic acid binds in a monodentate mode to a Ti_5c_ surface site (bi-deprMD). Differently from the linear structure of CO_2_ (molMD^CO_2_^), the bi-deprMD formic acid has an OCO angle of about 135°. This configuration is found to be a stable reaction intermediate during the photocatalytic oxidation of formic acid, as discussed in Sections 3.2.4 and 3.3.4.

When a water molecule is coadsorbed on the TiO_2_ surface, occupying the next neighboring Ti_5c_ site, the deprotonated formic acid can adsorb on the surface in a monodentate mode, forming a hydrogen bond with a H atom of the water molecule (deprMD + HB_w_). Moreover, TiO_2_ and TiO_2_^w^ are used in the following sections to distinguish between the dry and hydrated (with one adsorbed undissociated water molecule) anatase TiO_2_ (101) surfaces, respectively. Finally, after complete formic acid oxidation, the resulting reduced anatase TiO_2_ (101) surface is identified by TiO_2_–2OH and contains two extra electrons localized on Ti_6c_ atoms in the first and second layers of TiO_2_ beneath the protonated O_2c_ sites.

## 3 Results

### 3.1 Pathways of Formic Acid Oxidation

The oxidation of formic acid on the anatase TiO_2_ (101) surface was modeled through the optimization of several reaction intermediates suggested by previous experimental and theoretical studies.^29,31,32,34,40,42,48,60,70,71^ A plausible sequence of elementary steps and intermediates for formic acid oxidation is shown in Scheme 2a. In this scheme, the reaction initiates with the preliminary adsorption of formic acid on a surface Ti_5c_–O_2c_ pair to form molecularly bound FA, interacting with a Ti_5c_ site via the O atom in the carboxylic group and with a O_2c_ site via the H atom of the OH group. The O–H bond of molMD^FA^ is then broken, and the proton is transferred to the surface. The cleavage of the O–H bond from the molecularly adsorbed FA via H-abstraction by the O_2c_ leads to the formation of either a bidentate formate (deprBD) interacting with two Ti_5c_ atoms or a monodentate formate forming a H-bond with the dissociated proton (deprMD + HB). The bidentate formate species (with the coadsorbed dissociated proton) represents the most stable form of formic acid on TiO_2_ surfaces, as shown by many experimental and theoretical studies.^26,27,29–31,34,40,72^ The formation of CO_2_ takes place after cleavage of the C–H bond and transfer of the H atom to a neighboring surface O_2c_ atom.

**Scheme 2:**
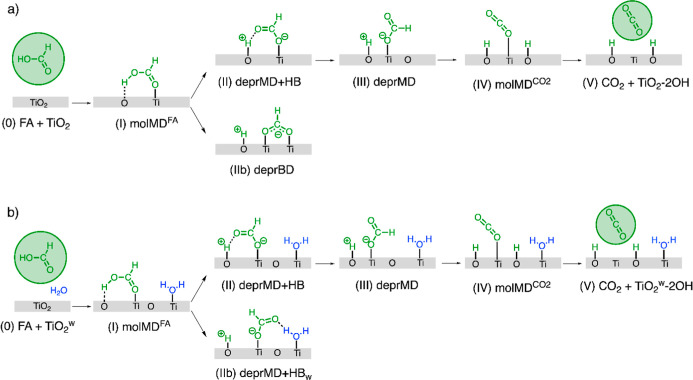
Mechanism of Formic Acid Oxidation on Anatase TiO_2_ (101) to Form CO_2_ in (a) Anhydrous and (b) Hydrated Conditions^a^

a Reaction intermediates are listed using Roman numerals and labeled according to the adsorption configuration of formic acid (green) on TiO_2_ (black). The gray bar represents the anatase (101) surface, where the Ti and O atoms indicate the undercoordinated Ti_5c_ and O_2c_ sites, respectively. Coadsorbed water molecules on the TiO_2_ surface are colored in blue. Dashed black lines indicate hydrogen bonds.

Although deprBD is more strongly bound to the TiO_2_ surface, both experiments and calculations found that a more reactive monodentate structure is required for the oxidation of formic acid.^28,33,40,44^ The monodentate structure can originate from rotation of the dissociated bidentate or from a molecularly adsorbed species (molMD^FA^) that loses its proton to the surface. The oxidation of FA starting from the stable deprBD structure requires a greater amount of energy in order to break one of the Ti–O covalent bonds.^34,70^ Indeed, previous studies have shown that monodentate formate is more reactive during formic acid oxidation, given the energy required to activate and break the Ti–O bond in the bidentate formate.^33,40,44,60^

When the anatase surface is hydrated, fewer surface sites are available for the adsorption of formic acid, favoring monodentate adsorption while hindering the bidentate configuration. In Scheme 2b, the reaction intermediates for the oxidation of formic acid in the presence of coadsorbed water are schematically illustrated. However, one of the carboxylate O atoms of the deprotonated formic acid can form a hydrogen bond with a water H atom, resulting in a stable monodentate (deprMD + HB_w_) adsorption mode. Compared to the formation of the deprBD state under dry conditions, the formation of deprMD + HB_w_ is reversible because the H-bond with the water molecule is much weaker than the strong covalent bond with the anatase surface.

### 3.2 Formic Acid Adsorption and Oxidation on Dry TiO_2_

#### 3.2.1 Formic Acid Adsorption on TiO_2_ in the (Singlet) Ground State

The molecular and dissociated adsorption geometries of FA on the bare anatase TiO_2_ (101) surface are detailed in Figure 1.^28,29,40–42,45,47,48,59,71^ In its molecular monodentate configuration (molMD^FA^), formic acid binds to a surface Ti_5c_ site through the carboxyl oxygen, while the hydroxyl group forms a hydrogen bond with a surface O_2c_ atom. Two possible configurations are identified: intra-pair, where the Ti_5c_ and O_2c_ sites belong to the same Ti–O bound surface pair; and inter-pair, where they belong to different Ti–O surface pairs not bound to each other. Dissociated formic acid generally adsorbs in a bridging bidentate configuration (deprBD), where both formate oxygen atoms are coordinated to two adjacent Ti_5c_ sites along the [010] direction, while the proton is transferred to a nearby O_2c_ atom. The specific configuration (intra-pair or inter-pair) for the deprBD intermediate is determined by the O_2c_ atom to which the proton is transferred.

**Figure 1:**
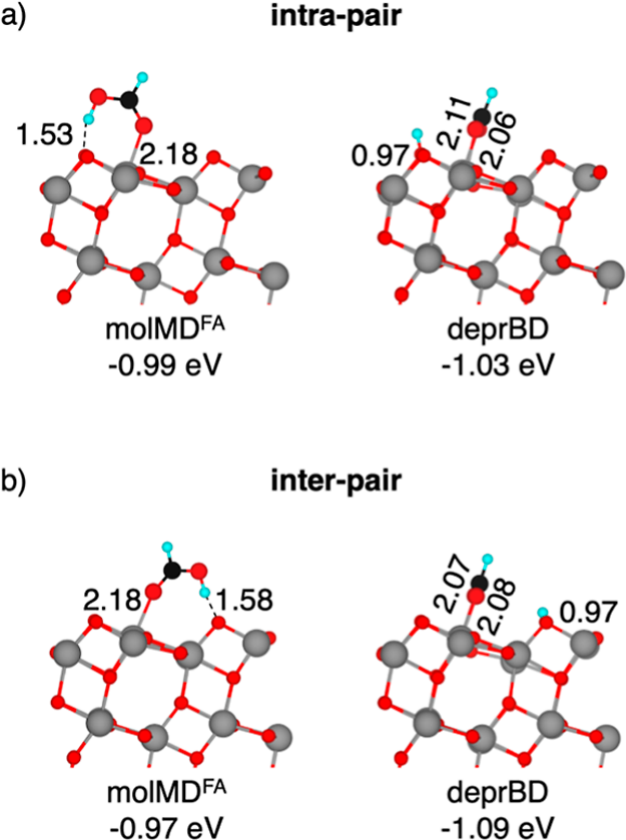
Structures of the molecular monodentate (molMD^FA^) and deprotonated bidentate (deprBD) (a) intra-pair and (b) inter-pair configurations of adsorbed FA on the anatase TiO_2_ (101) surface computed using the HSE06 density functional in the singlet ground state. Adsorption energies (in eV) and selected bond lengths (in Å) are reported. Cyan, black, red, and gray spheres represent H, C, O, and Ti atoms, respectively. For better visualization, FA spheres have been magnified.

Our computed structures for the molMD^FA^ and deprBD configurations are consistent with those reported in the literature.^30,34,47^ The calculated adsorption energies indicate that dissociative bidentate adsorption is favored, in line with previous experimental observations and DFT calculations.^31–34,40,47,48,73^ While the energy difference between inter-pair and intra-pair molMD^FA^ structures is minimal (−0.02 eV in favor of intra-pair), inter-pair deprBD configurations are −0.06 eV more favorable. The intra- and inter-pair molMD^FA^ adsorption structures serve as the starting points for two different reaction mechanisms of FA oxidation on TiO_2_, namely, the intra-pair and the inter-pair route.

#### 3.2.2 Thermal Oxidation of Formic Acid on TiO_2_

In this section, we report the thermal oxidation mechanism of FA on dry anatase TiO_2_ (101) starting from the most stable molMD^FA^ structure in the intra-pair configuration (Figure 2).

**Figure 2:**
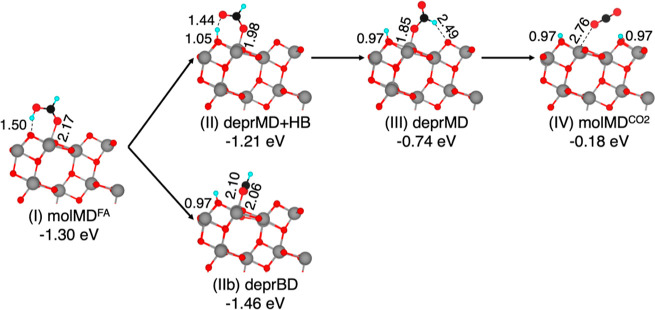
Structures of the intermediates of formic acid thermal oxidation along the intra-pair route on the anatase TiO_2_ (101) surface computed using the HSE06-D3 density functional. Adsorption energies (in eV) and relevant bond lengths (in Å) are reported. Cyan, black, red, and gray spheres represent H, C, O, and Ti atoms, respectively. For better visualization, FA spheres have been magnified.

Starting from the molMD^FA^ adsorption structure in Figure 2I, the initial step involves the transfer of the acid proton to a surface O_2c_ (deprMD + HB structure in Figure 2II). Upon deprotonation, the molecule in the deprMD + HB configuration moves closer to the TiO_2_ surface by shortening its carboxylic O to Ti bond by approximately 0.2 Å while maintaining a hydrogen bond with its dissociated proton. This step leads to a less favorable adsorption energy (−1.21 eV) compared to that of the molecular monodentate adsorption intermediate (−1.30 eV).

The subsequent rotation of the formate molecule, with the concerted breaking of the hydrogen bond, is also an endothermic step, resulting in the deprMD structure (Figure 2III). Here, the carboxylic O to Ti bond is further shortened by 0.13 Å and the adsorption energy is decreased to −0.74 eV.

The final step involves cleavage of the C–H bond, resulting in the formation of a weakly bound CO_2_ molecule (molMD^CO_2_^) in the last endothermic step before CO_2_ desorption. The CO_2_ molecule weakly interacts with a Ti surface site, and the two hydrogen atoms from the FA molecule are now bound to the nearest and second nearest O_2c_ surface sites, resulting in a high-energy intermediate with two excess electrons in the TiO_2_ surface. We also investigated whether releasing the close-shell constraint and allowing the reduced TiO_2_ surface with two unpaired electrons to form a triplet state could lower the total energy of the molMD^CO_2_^ structure (Figure 2IV). We found that the open-shell spin configuration is indeed more stable by −0.20 eV. The spin densities show the two electrons localized on Ti_6c_ atoms in the first and second TiO_2_ layers beneath the protonated O_2c_ sites.

In the above pathway, the deprBD configuration constitutes a stable but detrimental intermediate for the oxidation reaction (Figure 2IIb). Indeed, it generates a lateral path that significantly slows the oxidation reaction since the formation of the deprMD intermediate would require, in this case, breaking a strong covalent bond between a carboxylate O and a surface Ti_5c_.

The intermediates of the inter-pair pathway originating from the inter-pair molMD^FA^ are reported in the Supporting Information (Figure S2). This pathway is totally analogous to that for the intra-pair route (Figures 2 and S1), except for a small difference related to the formation of the (III) deprMD intermediate. To provide the FA molecule with the necessary freedom to rotate and form the (III) deprMD intermediate, it was necessary to move the dissociated proton to an O_2c_ surface site further away from the formate in order to prevent the reformation of the H-bond, leading to the (II) deprMD + HB intermediate.

#### 3.2.3 Formic Acid Adsorption on TiO_2_ in the Lowest Excited (Triplet) State

An accurate description of photocatalytic oxidation reactions, which take place in photogenerated excited states (S1), requires methods beyond conventional DFT, which is inherently a ground-state theory. For TiO_2_, however, several studies have found that it is reasonable to replace the photogenerated S1 excited state by the T1 triplet state,^14,15,23–25^ which is the lowest energy state for this spin multiplicity and, thus, can be correctly obtained by spin-constrained DFT calculations.^14,15^

Figure 3 shows the most stable formic acid adsorption geometries in the presence of a photoexcited electron–hole pair in the triplet state (yellow contours). For molecular monodentate FA, the spin distribution corresponding to the photogenerated hole is localized on a single surface O_2c_ atom, while the electron is delocalized on multiple Ti sites of the second layer, consistent with our results for pristine anatase TiO_2_ (101) in the excited triplet state as well as with a previous study.^15^ In the photoexcited triplet state, the intra-pair molMD^FA^ and deprBD configurations are more stable than the corresponding inter-pair ones by −0.02 eV and −0.04 eV, respectively.

**Figure 3:**
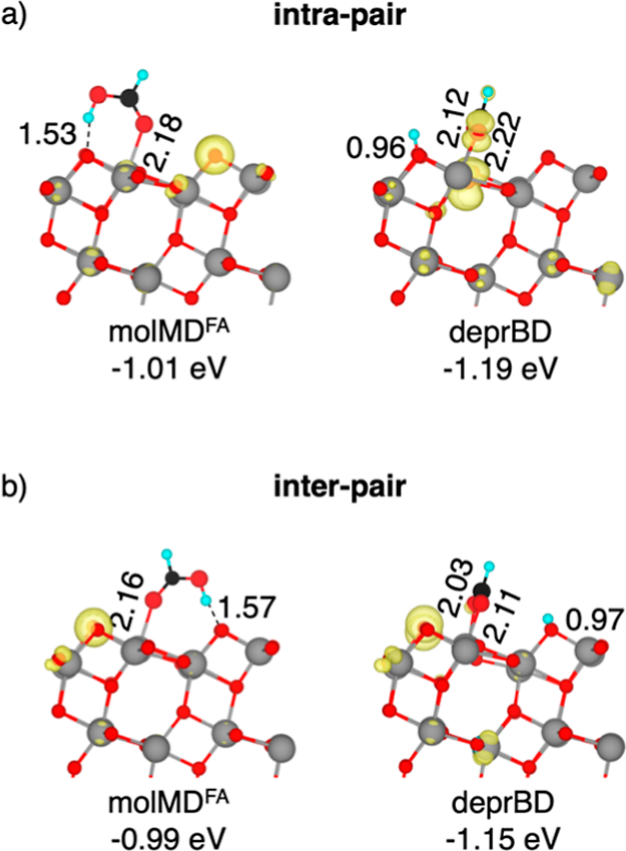
Structures of the molecular monodentate (molMD^FA^) and deprotonated bidentate (deprBD) (a) intra-pair and (b) inter-pair configurations of adsorbed FA on the anatase TiO_2_ (101) surface computed using the HSE06 density functional in the photoexcited triplet state. Adsorption energies (in eV) and selected bond lengths (in Å) are reported. Cyan, black, red, and gray spheres represent H, C, O, and Ti atoms, respectively. Clouds of spin localization have been plotted in yellow with an isovalue of 0.005 au using VESTA visualization software. For better visualization, FA spheres have been magnified.

The intra-pair deprBD structure displays a different spin distribution, with 23% of the spin localized on one carboxylic oxygen, causing a distortion in the adsorption structure symmetry compared to the singlet ground state. The remaining hole density (72%) is localized on a surface O_3c_ atom underneath the formate, with a distribution oriented toward the carboxylic oxygen. Indeed, formic acid is typically considered a good hole scavenger in photocatalysis.^30,70^ Our HSE06 calculations thus indicate partial hole delocalization between the adsorbed formic acid and a surface oxygen atom of the TiO_2_ surface, a feature that was similarly observed by Ji and Luo.^60,70^ Although this bidentate configuration enables effective hole localization, the subsequent oxidation reaction is hindered by the strong covalent bonds between the carboxylate O atoms and the surface Ti sites, which restrict the adsorbate mobility, which is essential for the oxidation process to proceed efficiently.

Results for several additional configurations of proton adsorption and charge localization are reported in Figure S3 in the Supporting Information. Particularly interesting is the intra-pair deprBD structure in Figure S3a, which is energetically degenerate with that in Figure 3 (−1.20 eV vs −1.19 eV, respectively) but has a different spin distribution. In the deprBD structure in Figure S3a, the photogenerated electron localizes on the undercoordinated surface Ti atom, binding one carboxylic oxygen while simultaneously bridging to the undercoordinated intra-pair O surface site used for proton adsorption. Even though the two most stable deprBD intra-pair structures are isoenergetic, the presence of the photoexcited electron localized at a surface Ti_5c_ directly involved with binding the organic molecule hinders the hole localization on the formate itself. Only when the electron is deeper into the TiO_2_ layers does successful trapping of the hole on one O atom of formate takes place.

#### 3.2.4 Photocatalytic Oxidation of Formic Acid on TiO_2_

The photocatalytic oxidation mechanism of FA on dry anatase TiO_2_ (101) is investigated starting from the most stable intra-pair molMD^FA^ structure. The most important configurations for the photocatalytic oxidation are those with the hole localized at surface sites since they can interact with the adsorbed molecule. The calculated intermediates for the FA oxidation in the photoexcited triplet state are shown with structural details in Figure 4.

**Figure 4:**
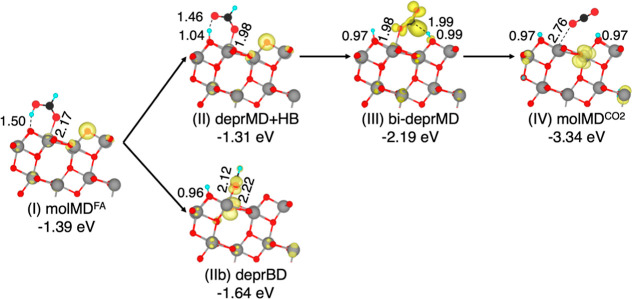
Structures of the intermediates of formic acid photocatalytic oxidation along the intra-pair route on the anatase TiO_2_ (101) surface computed using the HSE06-D3 density functional. Adsorption energies (in eV) and relevant bond lengths (in Å) are reported. Cyan, black, red, and gray spheres represent H, C, O, and Ti atoms, respectively. Clouds of spin localization have been plotted in yellow with an isovalue of 0.005 au using VESTA visualization software. For better visualization, FA spheres have been magnified.

The first step of the reaction involves the proton transfer from the adsorbed FA to the TiO_2_ surface (deprMD + HB in Figure 4II), resulting in a shorter carboxylic O to Ti bond length and less negative adsorption energy (−1.31 eV) than the adsorption energy of the molMD^FA^ structure (−1.39 eV). Similar to the reaction in the ground state, the formation of the deprMD + HB intermediate in the photoexcited state is endothermic by 0.08 eV. The deprMD + HB structure in the photoexcited state exhibits minimal changes in geometry and adsorption energy compared to its singlet ground state counterpart. The calculated spin distribution also remains unchanged from that of the molMD^FA^ structure, with the hole localized on the surface O_2c_ and the electron delocalized across multiple Ti sites in the second layer.

The subsequent rotation of monodentate formate in the photoexcited state (bi-deprMD in Figure 4III) differs significantly from that of the ground state intermediate (deprMD in Figure 2III). This rotation entails the concerted breaking of the O–H hydrogen bond and cleavage of the C–H bond, resulting in an exothermic step. After formate rotation, the C–H group moves close enough to the hole-bearing O_2c_ to facilitate transfer of the hole to the adsorbed molecule in the bi-deprMD intermediate (82% of localized spin on the adsorbate). The hole transfer is associated with a proton transfer from the adsorbed molecule to the surface. Therefore, while the hole is transferred to the adsorbate, the second proton of FA moves to the surface, resulting in a bideprotonated molecule that binds with one O atom in a monodentate mode (bi-deprMD). This process ends up in a very stable intermediate with an adsorption energy of −2.19 eV. Moreover, the C–H bond cleavage observed in the presence of a photogenerated hole proceeds via a homolytic mechanism and therefore should not be directly influenced by pH. Although the formic acid molecule has lost both protons, it retains its typical OCO formate angle, and the oxidation to the linear CO_2_ structure is achieved in the subsequent highly exothermic step. The final intermediate, molMD^CO_2_^ (Figure 4IV), marks the formation of CO_2_ through the complete oxidation of the organic molecule and the reduction of TiO_2_ by two electrons localized on the Ti_6c_ sites.

These results strongly suggest that the photogenerated hole predominantly mediates C–H bond cleavage, favoring CO_2_ formation on the anatase (101) surface. The spin densities indicate that the electron localizes on the Ti_6c_ atoms in the second layer beneath the protonated O_2c_ site. The observed intermediates show that the photocatalytic oxidation of formic acid takes place through an electron transfer after C–H breaking in the presence of a photogenerated hole localized on the TiO_2_ surface. A similar C–H breaking mechanism has been previously reported by Migani and Blancafort for the photocatalytic oxidation of methanol on rutile TiO_2_.^24^

As found for the reaction in the ground singlet state, the deprBD configuration constitutes a stable but detrimental intermediate for the oxidation reaction (Figure 4IIb). The strong covalent bonds between carboxylate O of deprBD and surface Ti_5c_ require high energy to be broken to form the bi-deprMD reaction intermediate; thus, this configuration significantly slows down the PCO.

The reaction intermediates of the photocatalytic oxidation route originating from the inter-pair molecular molMD^FA^ adsorption configuration are reported in the Supporting Information (Figure S5). The inter-pair intermediates in the photoexcited triplet state (Figure S5) follow the same oxidation mechanism as that observed for the intra-pair photocatalytic reaction route (Figures 4 and S4).

### 3.3 Role of Water on Formic Acid Adsorption and Oxidation on TiO_2_

#### 3.3.1 Formic Acid Adsorption on Hydrated TiO_2_ in the (Singlet) Ground State

The adsorption configurations of formic acid on hydrated anatase (101) are similar to those on the dry surface (Figure 5). However, the presence of coadsorbed water molecules can change the relative stabilities of the adsorption modes.

**Figure 5:**
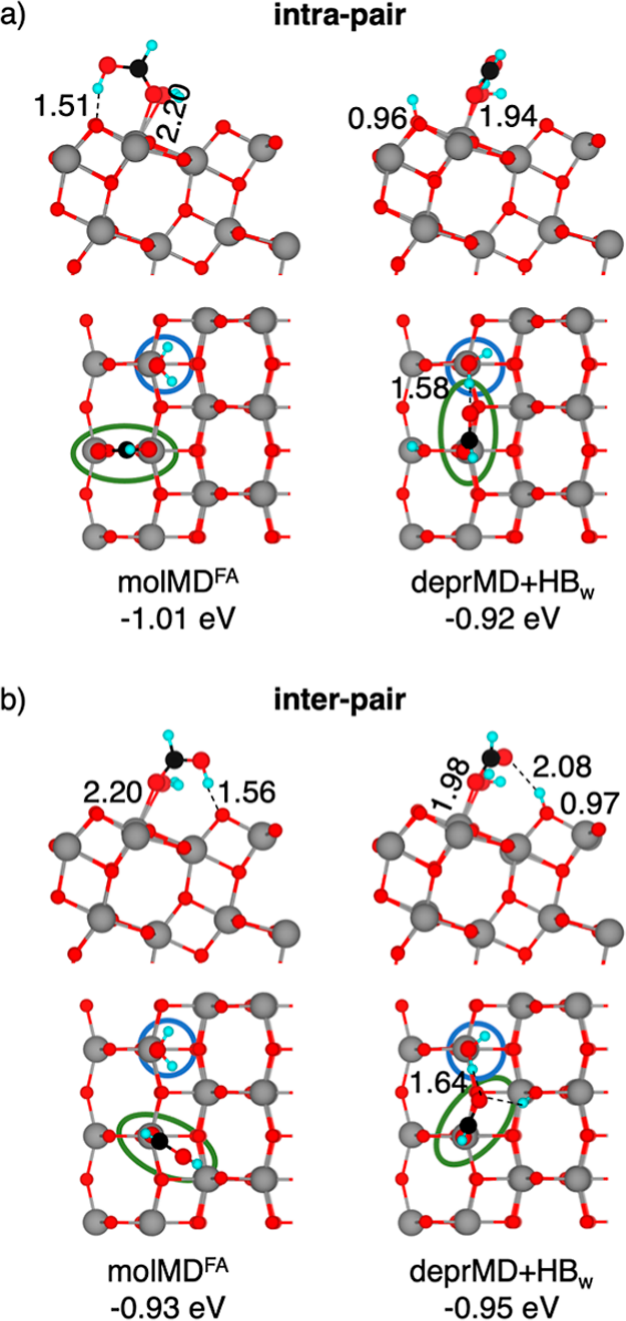
Structures of the molecular (molMD^FA^) and deprotonated monodentate (deprMD + HB_w_) (a) intra-pair and (b) inter-pair configurations of adsorbed FA on the anatase TiO_2_ (101) surface in the presence of coadsorbed water molecules computed using the HSE06 density functional in the singlet ground state. Adsorption energies (in eV) and selected bond lengths (in Å) are reported. Cyan, black, red, and gray spheres represent H, C, O, and Ti atoms, respectively. On the top view, showing the top layer of TiO_2_, blue and green circles highlight the position of adsorbed water and formic acid molecules on the surface, respectively. For better visualization, FA spheres have been magnified.

The presence of a water molecule does not affect the adsorption geometry or adsorption energy of the intra-pair molMD^FA^ structure (Figure 5a), which is −1.01 eV on the hydrated surface compared to −0.99 eV on the anhydrous surface. Inter-pair molecular monodentate adsorption results instead in two different configurations depending on which O_2c_ surface site the proton is transferred to: the O_2c_ site closer to the water molecule that is already involved in a H-bond (molMD^FA^ in Figure S6) or the O_2c_ site further away from the water molecule (molMD^FA^ in Figure 5b). Nonetheless, the adsorption energies of these molecular adsorption modes are similar and slightly less negative than those of the intra-pair structure (by +0.08 eV), as previously seen also for the dry surface (+0.02 eV).

Adsorbed water may hinder the formation of the deprBD structure due to the occupation of one of the two neighboring adsorption sites required to form a bidentate structure. However, a new deprotonated monodentate adsorption mode deprMD + HB_w_ can occur, where one of the formic acid’s O atoms binds to a surface Ti atom and the other forms a H-bond with the water molecule (Figure 5a,b). The most stable dissociated deprMD + HB_w_ structure is an inter-pair adsorption mode (adsorption energy of −0.95 eV), where the formic acid O atom forms hydrogen bonds with both the water molecule and the dissociated proton on a nearby O_2c_ site.

#### 3.3.2 Thermal Oxidation of Formic Acid on Hydrated TiO_2_

In this section, we investigate the thermal oxidation mechanism of FA on hydrated anatase TiO_2_ (101) starting from the most stable intra-pair molMD^FA^ structure, while the reaction intermediates originating from the inter-pair configurations are reported in the Supporting Information (Figures S8 and S9).

In the first step, molMD^FA^ (Figure 6I) transfers its acidic proton to a surface O_2c_ atom, resulting in deprMD + HB (Figure 6II). This process, similar to that observed on the anhydrous TiO_2_ surface, shortens the carboxylic O to Ti bond by 0.19 Å, leading to a less favorable adsorption energy (−1.31 eV) with respect to that of the molMD^FA^ structure (−1.34 eV).

**Figure 6:**
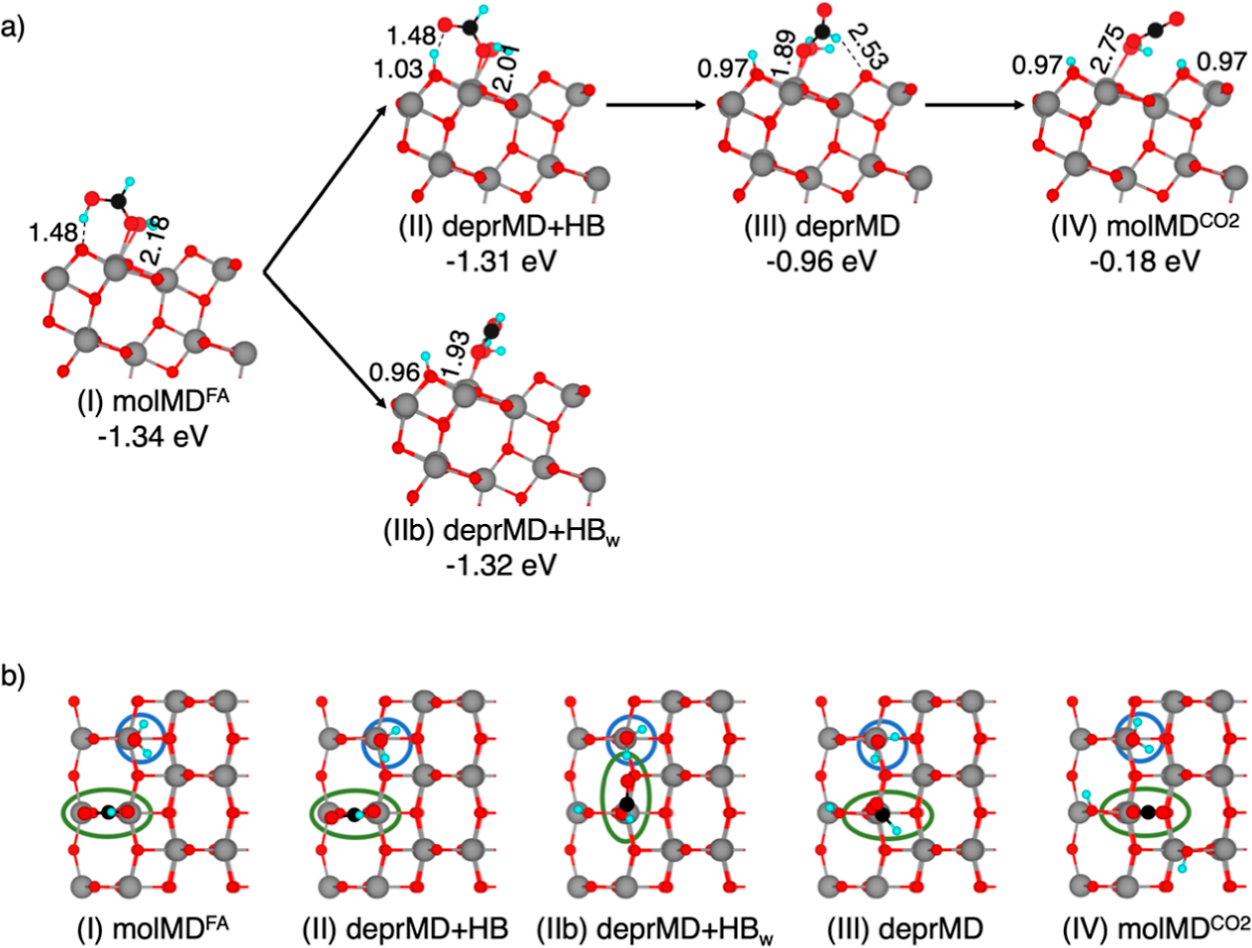
Structures of the intermediates of formic acid thermal oxidation along the intra-pair route on the anatase TiO_2_ (101) surface in the presence of coadsorbed water molecules computed using the HSE06-D3 density functional. On the side view (a), showing the first two layers of TiO_2_, adsorption energies (in eV) and relevant bond lengths (in Å) are reported. On the top view (b), showing the top layer of TiO_2_, blue and green circles highlight the position of adsorbed water and formic acid molecules on the surface, respectively. Cyan, black, red, and gray spheres represent H, C, O, and Ti atoms, respectively. For better visualization, FA spheres have been magnified.

Subsequently, the formate in deprMD + HB can break its H-bond with the surface hydroxyl and form a new H-bond with a water H, leading to the formation of the deprMD + HB_w_ intermediate (Figure 6IIb). The deprMD + HB and deprMD + HB_w_ intermediates are isoenergetic, suggesting a rapid and facile interconversion between them. However, it is only after all hydrogen bonds are disrupted that the monodentate formate can fully rotate to bring its (C-bound) H atom into the proximity of a surface O_2c_, facilitating the final cleavage of the C–H bond. The concerted breaking of the hydrogen bonds and formation of a weak interaction between the H of the formate and the surface O_2c_ result in a stable intermediate (deprMD in Figure 6III) with an adsorption energy of −0.96 eV. Finally, after formic acid has lost both protons, CO_2_ is formed on the anatase surface (Figure 6IV) in an endothermic process. In the last step of the reaction mechanism, once again, formic acid is completely oxidized to CO_2_, with the anatase surface being reduced by two unpaired electrons.

The intermediates of the FA thermal oxidation originating from the molecular inter-pair adsorption configurations of formic acid are shown in Figures S8 and S9. The behaviors of the molMD^FA^, deprMD + HB, and deprMD + HB_w_ intermediates are very similar to those shown in Figure 6 (and Figure S7). The formation of a stable deprMD intermediate was however unsuccessful for the inter-pair route since the close proximity of the water H and the dissociated proton on the surface caused the formate molecule to form H-bonds with either one of these species.

#### 3.3.3 Formic Acid Adsorption on Hydrated TiO_2_ in the Lowest Excited (Triplet) State

Figure 7 shows the most stable adsorption geometries of formic acid on the hydrated anatase surface in the photoexcited state. The presence of a coadsorbed water molecule does not significantly affect the hole localization on the surface O_2c_ atoms nor the state of the electron, which remains delocalized on Ti_6c_ atoms of the second layer. In the photoexcited triplet state, the intra-pair configuration is more stable than the inter-pair one by −0.02 eV for both the molMD^FA^ and deprBD structures.

**Figure 7:**
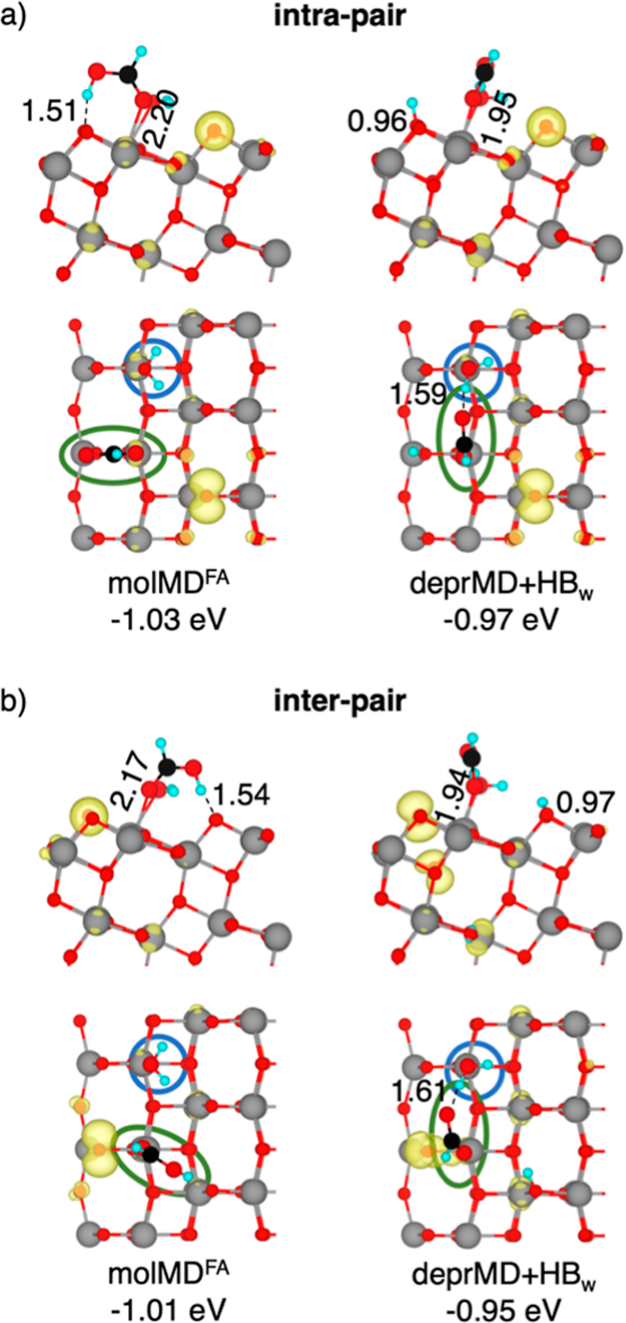
Structures of the molecular (molMD^FA^) and deprotonated monodentate (deprMD + HB_w_) (a) intra-pair and (b) inter-pair configurations of adsorbed FA on the anatase TiO_2_ (101) surface in the presence of coadsorbed water molecules computed using the HSE06 density functional in the photoexcited triplet state. Adsorption energies (in eV) and selected bond lengths (in Å) are reported. Cyan, black, red, and gray spheres represent H, C, O, and Ti atoms, respectively. On the top view, showing the top layer of TiO_2_, blue and green circles highlight the position of adsorbed water and formic acid molecules on the surface, respectively. Clouds of spin localization have been plotted in yellow with an isovalue of 0.005 au using VESTA visualization software. For better visualization, FA spheres have been magnified.

#### 3.3.4 Photocatalytic Oxidation of Formic Acid on Hydrated TiO_2_

Figure 8 shows the intermediates of the photocatalytic oxidation of formic acid on hydrated anatase TiO_2_ (101) along the intra-pair route that starts from the most stable intra-pair molMD^FA^ structure. The intermediates of the photocatalytic oxidation route originating from the inter-pair molMD^FA^ configuration are reported in the Supporting Information (Figure S12).

**Figure 8:**
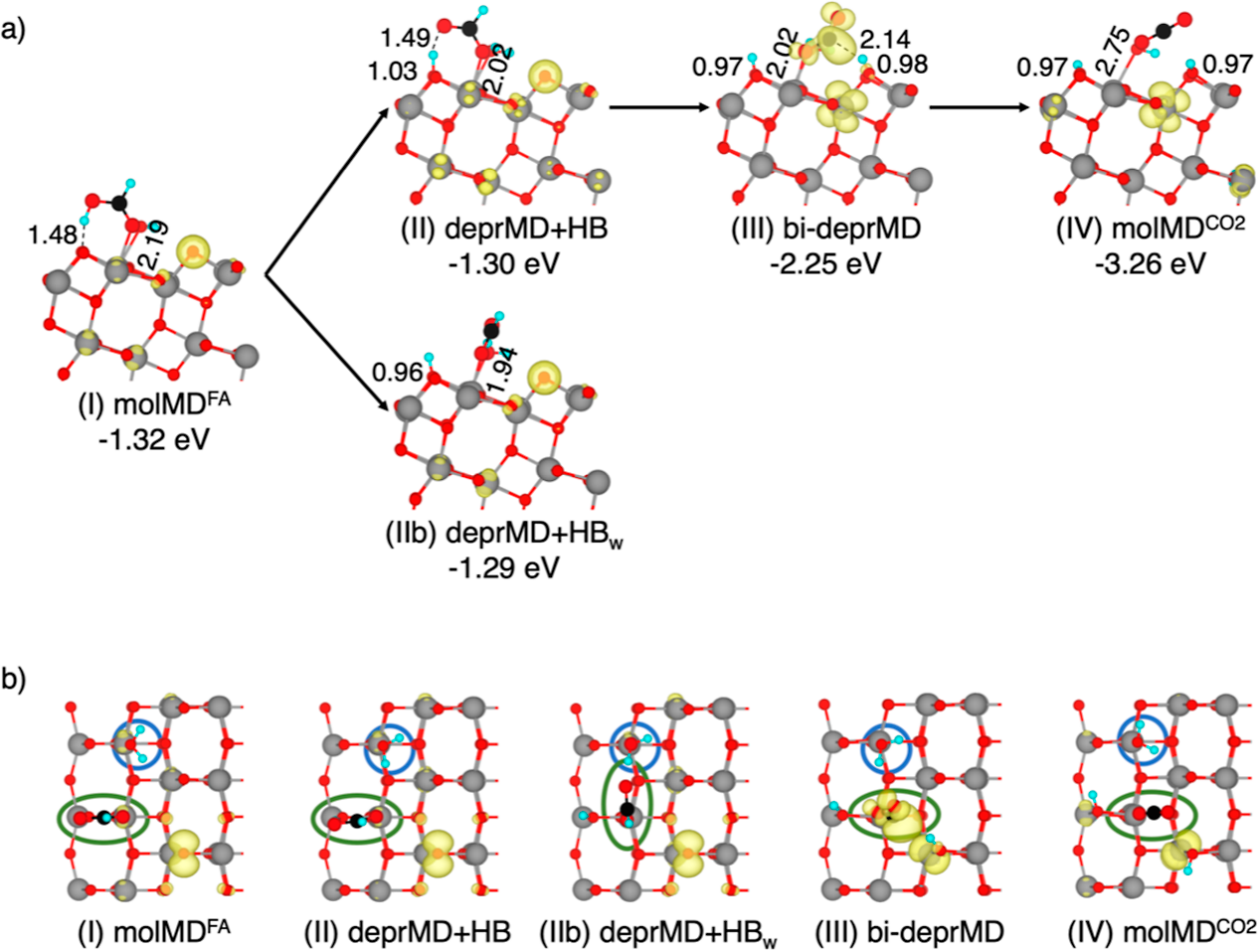
Structures of the intermediates of formic acid photocatalytic oxidation along the intra-pair route on the anatase TiO_2_ (101) surface in the presence of coadsorbed water molecules computed using the HSE06-D3 density functional. On the side view (a), showing the first two layers of TiO_2_, adsorption energies (in eV) and relevant bond lengths (in Å) are reported. On the top view (b), showing the first layer of TiO_2_, blue and green circles highlight the position of adsorbed water and formic acid molecules on the surface, respectively. Cyan, black, red, and gray spheres represent H, C, O, and Ti atoms, respectively. Clouds of spin localization have been plotted in yellow with an isovalue of 0.005 au using VESTA visualization software. For better visualization, FA spheres have been magnified.

The initial proton transfer from molMD^FA^ (Figure 8I) to the TiO_2_ surface leads to deprMD + HB (Figure 8II), which has a shorter carboxylic O to Ti bond (by 0.17 Å) and a slightly less favorable adsorption energy (−1.30 eV) with respect to molMD^FA^ (−1.32 eV). The formate can rotate, and the proximity of a water molecule facilitates the formation of a new hydrogen bond between the deprotonated O atom of the formate and a water H, resulting in the deprMD + HB_w_ intermediate (Figure 8IIb), with an adsorption energy of −1.29 eV. The path from molMD^FA^ to deprMD + HB and deprMD + HB_w_ in the presence of water is almost barrierless in the photoexcited state, indicating a rapid and facile interconversion between these structures.

The subsequent step is pivotal for the complete oxidation of formic acid. After all hydrogen bonds are broken, the monodentate formate can rotate to bring its C-bound H atom close to the surface O_2c_ where the hole is localized. The proximity of the hole facilitates the cleavage of the C–H bond, resulting in the bi-deprMD structure (Figure 8III) that has a significantly more negative adsorption energy of −2.25 eV. The spin plot reveals that the hole is now localized on the adsorbate (86%). However, intermediates with the hole localized at a different surface O_2c_ can also form (Figure S11) without any significant differences in the reaction mechanism. In this step, FA is not completely oxidized yet, and the CO_2_ structure is achieved only with the final reduction of the TiO_2_ surface by two unpaired electrons. The spin densities show these two electrons localized on Ti_6c_ atoms in the first and second TiO_2_ layers beneath the protonated O_2c_ sites.

## 4 Discussion

As an overview of the effects of photogenerated electron–hole pairs and coadsorbed water molecules on FA (photo)­oxidation, Figure 9 shows the calculated Gibbs free adsorption energies of the reaction intermediates along the various intra-pair paths investigated in this work. While the energy values reported so far are adsorption energies at *T* = 0 K, we also calculated the Gibbs free adsorption energies at *T* = 298.15 K by inclusion of the zero-point energy values and entropic contribution, as detailed in the Computational Methods section (Section 2.1). Gibbs free energy values do not change the qualitative features of the thermal oxidation energy profile under dry or hydrated conditions (Figure 9) compared with the *T* = 0 K energy profiles (Figure S13). Adsorption free energies for all investigated intermediates are systematically shifted to slightly more positive values (Tables 1 and S1).

**Figure 9:**
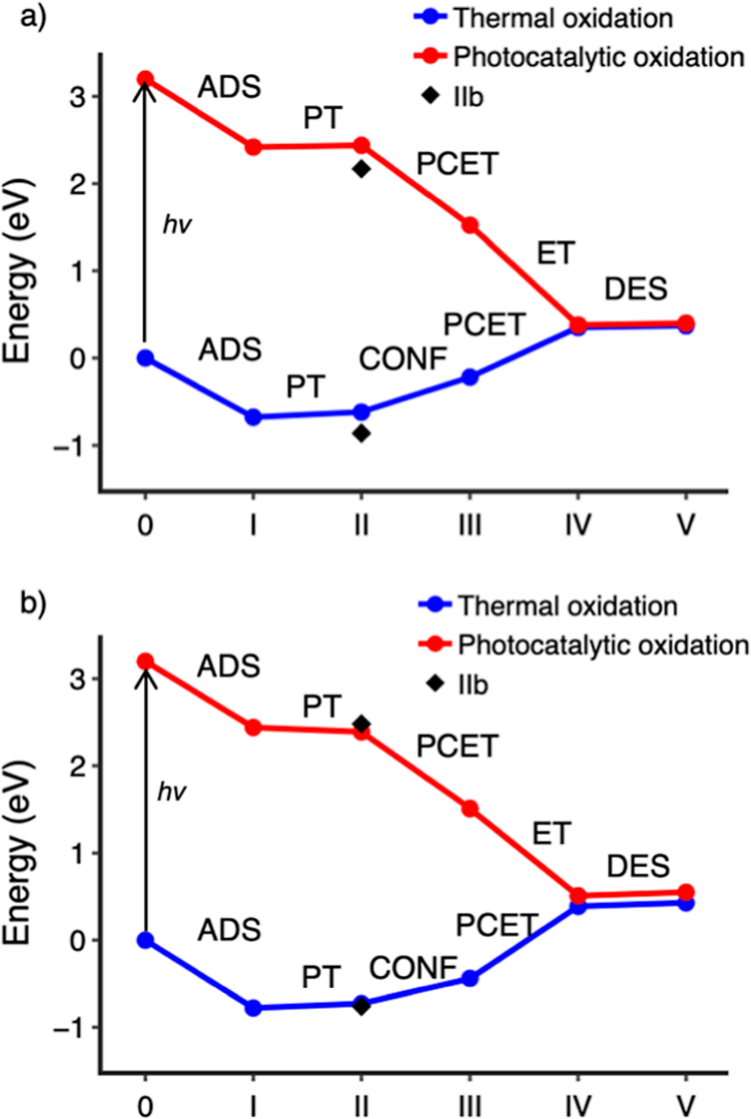
Plots of the Gibbs free adsorption energy (as defined in eq 3) for the intermediates of the formic acid thermal (blue) and photocatalytic (red) oxidation along the intra-pair route on anatase TiO_2_ (101) in (a) dry and (b) hydrated conditions, calculated with HSE06-D3. The excitation cost to start the photocatalytic reaction is highlighted by a black arrow. The zero-energy value is the energy of the noninteracting reference anatase (101) slab and gas phase formic acid molecule. Diamonds indicate the adsorption energy of the (IIb) intermediate (see the text). Each step is labeled according to its nature: reactant adsorption (ADS), deprotonation (PT), proton-coupled electron transfer (PCET), geometry configuration rotation (CONF), and product desorption (DES).

**Table 1:** Calculated Adsorption Free Energy Values (Obtained Using HSE06-D3 and the Definition in Equation 3) for the Intermediates of the Formic Acid Thermal and Photocatalytic Oxidation Reaction along the Intra-pair Route on Anatase TiO_2_ (101) in Anhydrous and Hydrated Conditions^a^

	*G*_ads_ (eV) anhydrous conditions		*G*_ads_ (eV) hydrated conditions	
intra-pair intermediates	thermal	photocatalytic	thermal	photocatalytic
0	0.00	0.00	0.00	0.00
I	–0.68	–0.78	–0.78	–0.76
II	–0.62	–0.76	–0.73	–0.81
IIb	–0.86	–1.03	–0.76	–0.72
III	–0.22	–1.68	–0.45	–1.69
IV	+0.35	–2.82	+0.39	–2.69
V	+0.37	–2.80	+0.43	–2.65

a For each system, the zero energy is the sum of the energy of the corresponding anatase (101) slab and the energy of isolated gas phase formic acid.

Figure 9 clearly shows the opposite overall endothermic and exothermic trends of the reactions in the ground and photoexcited states, respectively. Considering first anhydrous conditions (Figure 9a), the energy difference between (IIb) deprBD and (II) deprMD + HB exhibits minimal variation between the singlet ground state and the photoexcited triplet state (0.15 and 0.28 eV, respectively, from Table 1). This indicates that the presence of a photoexcited hole does not affect the initial steps of the oxidation reaction. Instead, the role of the photogenerated hole is essential for cleavage of the C–H bond and the complete reduction of FA to CO_2_. As reported in the previous sections, the proximity between the hole localized at the surface O_2c_ and the H atom of the C–H bond favors the spontaneous cleavage of the latter, forming a stable bi-deprMD intermediate that subsequently evolves into the even more stable molMD^CO_2_^ intermediate. The concerted proton-coupled hole transfer process observed for the excited state is, therefore, responsible for the exothermic photocatalytic oxidation of formic acid on TiO_2_.

A qualitative kinetic analysis based on literature data supports the plausibility of the proposed reaction pathway. First, the transformation from the molecularly adsorbed formic acid (molMD^FA^) to the more stable deprotonated bidentate form (deprBD) has been reported to involve a moderate energy barrier of 0.30 eV^34^ or 0.35 eV.^70^ In contrast, the conversion from molMD^FA^ to the deprotonated deprMD + HB intermediate is barrierless,^34,48^ which highlights the kinetic favorability of this path. The subsequent evolution from deprMD + HB to deprMD involves cleavage of a hydrogen bond. Since this step entails breaking the same hydrogen bond required in the molMD^FA^ to deprBD pathway, it can be assumed to proceed with a comparable energy barrier (0.3 eV), although the final adsorption configuration differs. A higher energy barrier characterizes the formation of the bidentate deprotonated species bi-deprMD, with a reported transition state at 1.03 eV for a similar step,^33^ suggesting that this species is less kinetically accessible under typical thermal conditions. However, our results show that the transformation to the bi-deprMD intermediate becomes barrierless (proton transfer takes place during geometry optimization) in the photoexcited path, driven by the presence of a hole localized at a neighboring surface oxygen atom, which favors the coupled electron/proton transfer. Finally, the formation of the final products CO_2_ + TiO_2_–2OH from the molMD^CO_2_^ intermediate involves an energy barrier of approximately 0.35 eV, as inferred from studies of CO_2_ diffusion on TiO_2_ surfaces.^74^ Together, these kinetic considerations, supported by the literature and our own calculations, reinforce the conclusions based on thermodynamic results presented in this work and highlight the crucial role of the photoexcitation in lowering key barriers along the reaction pathway.

The trends for the hydrated surface are similar to those for the anhydrous case (Figure 9b). However, the presence of coadsorbed water molecules occupying Ti_5c_ adsorption sites limits the possibilities of bidentate adsorption (deprBD) in favor of the deprMD + HB_w_ configuration, where a carboxylic O forms a H-bond with adsorbed water. The weaker H-bond between a carboxylic O atom and a water H atom in deprMD + HB_w_, compared to the covalent bond between the carboxylic O atom and a surface Ti in deprBD, allows for faster interconversion with the monodentate molMD^FA^ and deprMD + HB intermediates. Moreover, the energy difference between deprMD + HB and deprMD + HB_w_ (0.02 eV) in hydrated conditions (blue line in Figure 9b) is way smaller than the one between deprMD + HB and deprBD (0.15 eV from Table 1) in anhydrous conditions (blue line in Figure 9a). This is also observed in the photoexcited triplet state (red lines in Figure 9 and Table 1) and shows the important role of water in favoring monodentate relative to bidentate adsorption and hindering the detrimental lateral path observed under anhydrous conditions.

Our results indicate that the coadsorption of water has a substantial influence on the adsorption configurations at the TiO_2_ surface, consistent with a previous study by Medlin et al.^62^ that demonstrated how different adsorbates can modulate the electronic structure and band edges of anatase TiO_2_. Specifically, Medlin et al. showed that monodentate formate adsorption lowers the conduction band (CB) edge below the O_2_/O_2_^–^ reduction potential, while the presence of coadsorbed water shifts it back above this threshold.^62^ In agreement with these findings, we observe that in the deprMD formic acid adsorption structure, both the valence band (VB) and conduction band edges shift downward, corresponding to a decrease in the reduction potential and an increase in the oxidation potential. However, when water is coadsorbed, these band edge positions are restored to values similar to those of the bare TiO_2_ slab (see Figure S14). This recovery enhances the surface’s reduction potential. Moreover, coadsorption of water also raises the VB edge toward the value of the clean TiO_2_ surface, at a level sufficient to drive the oxidation of formate. Together, these observations underline the critical role of water in stabilizing adsorbate structures and modulating the electronic structure to favor the efficient photocatalytic activity.

Similar trends are also reported for the oxidation of formic acid on TiO_2_ starting from the inter-pair adsorption configuration (Figure S14 and Table S2). The presence of coadsorbed water appears to slow down the thermal oxidation along the inter-pair route since the oxidation of formic acid to CO_2_ requires breaking the H-bonds between formate and surface OH groups or adsorbed water molecules. However, in the excited triplet state, these barriers are easily overcome as the hole transfer facilitates the C–H bond breaking, enabling the reaction to proceed.

## 5 Conclusions

We have investigated the adsorption and (photo)­catalytic oxidation of formic acid on dry and hydrated anatase TiO_2_ (101) surfaces using hybrid DFT/HSE06 calculations in the ground and lowest triplet excited states to simulate the thermal and photocatalytic reaction, respectively. For the latter, the open-shell triplet spin configuration was employed as a simplified approach to model the reaction intermediates in the photoexcited state.

Our results show that under dry conditions, formic acid preferentially adsorbs in a deprotonated bidentate mode on the anatase (101) surface, in agreement with previous studies.^31–34,40,47,73^ However, this stable bidentate structure requires a considerable amount of energy to break one of its Ti–O covalent bonds and transform into an active intermediate for the oxidation reaction and is thus detrimental to formic acid oxidation. This reaction is endothermic in the ground state, with the largest energy difference associated with the rotation of the monodentate formate to bring the C-bound H atom closer to the anatase surface prior to C–H bond cleavage (deprMD intermediate). In contrast, our results show that the presence of a photoexcited electron–hole pair dramatically alters this picture: the oxidation becomes a highly exothermic process, and the photogenerated hole, when localized at a nearby bridging oxygen (O_2c_) site, plays a key role in promoting C–H bond cleavage and facilitating CO_2_ formation. Our work thus provides direct evidence of hole-mediated activation pathways for surface-bound intermediates in TiO_2_ formic acid photocatalytic oxidation.

The presence of coadsorbed water on the TiO_2_ surface can hinder the deprotonated bidentate adsorption of formic acid, promoting instead the formation of a H-bond between the O atom of the formic acid and the H atom of water. This effect inhibits the detrimental formation of the bidentate binding modes during photo-oxidation and thus likely contributes to the experimentally observed enhancement in the rate of photocatalytic oxidation of formic acid in the presence of water. Moreover, the presence of coadsorbed water molecules lowers the energy barrier for the interconversion between the various monodentate deprotonated adsorption geometries of formic acid on the anatase (101) surface during the oxidation reaction. This reduction in energy facilitates the formation of the reactive deprMD + HB intermediate, thereby improving the reaction rate. By capturing the thermodynamic stabilization induced by water, our study provides a quantitative explanation for the experimentally observed enhancement in formic acid photo-oxidation rates under wet conditions.

The detailed analysis of the reaction path presented in this work yields valuable information for understanding the photocatalytic oxidation mechanism of formic acid on TiO_2_. In particular, it clearly shows why the photogenerated hole plays a crucial role in the C–H bond cleavage step to form CO_2_ and unveils the effect of coadsorbed water in favoring the formic acid monodentate adsorption and inhibiting the detrimental bidentate anchoring mode.

## Supplementary Material



## Notes

Structures, energy profiles, and table of calculated adsorption energies of the formic acid thermal and photocatalytic oxidation intermediates along the inter-pair route on the anatase TiO_2_ (101) surface in anhydrous and hydrated conditions; additional structures of low-energy adsorption configurations of formic acid on the anhydrous anatase TiO_2_ (101) surface in the triplet photoexcited state; structures of formic acid photocatalytic oxidation intermediates along the alternative intra-pair route on the hydrated anatase TiO_2_ (101) surface; and projected density of states (PDOS) for the anatase TiO_2_ (101) surface slab bare and with one formic acid molecule adsorbed in the deprMD configuration without and with coadsorbed water molecules (PDF)
