# Supplementary material for: Pathways of Photocatalytic Oxidation of Formic Acid on Dry and Hydrated Anatase TiO2 Surfaces
Source: ACS Catal. 2025 Jun 18;15(13):11487–501. doi: 10.1021/acscatal.5c01848 (PMC12235595; doi:10.1021/acscatal.5c01848)
Supplement: Supplementary file 1 [file cs5c01848_si_001.pdf]

Supporting Information

Pathways of Photocatalytic Oxidation of Formic Acid on Dry and Hydrated Anatase TiO_2 Surfaces

Chiara Daldossi[1], Cristiana Di Valentin[1,*] & Annabella Selloni[2,*]

[1] Department of Materials Science, University of Milano-Bicocca, via R. Cozzi 55, 20125 Milano, Italy

[2] Department of Chemistry, Princeton University, Princeton, New Jersey 08544, United States

[*] Corresponding authors: cristiana.divalentin@unimib.it; aselloni@princeton.edu

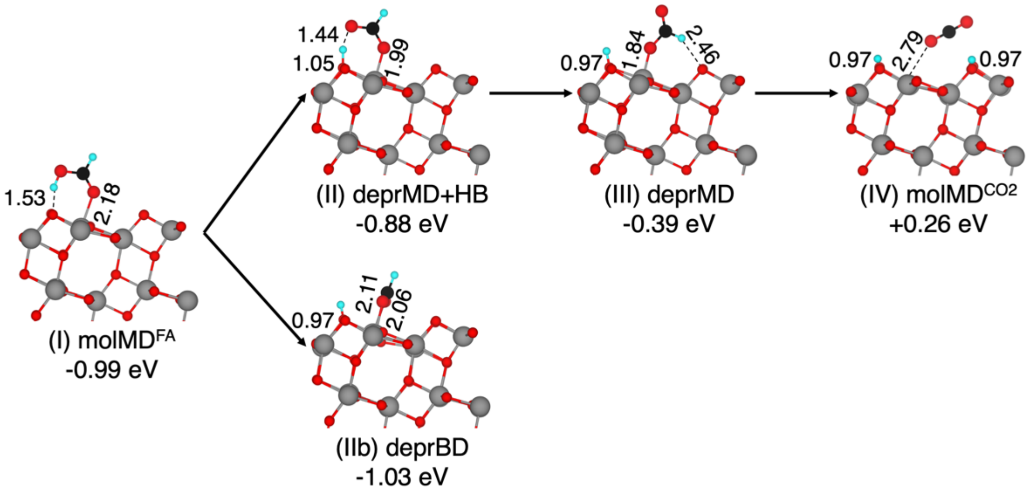

*Figure S1. Structures of formic acid thermal oxidation intermediates along the **intra**-pair route on the anatase TiO_2 (101) surface computed using the HSE06 density functional. Adsorption energies (in eV) and relevant bond lengths (in Å) are reported. Cyan, black, red, and grey spheres represent H, C, O, and Ti atoms, respectively. For better visualization, FA spheres have been magnified.*

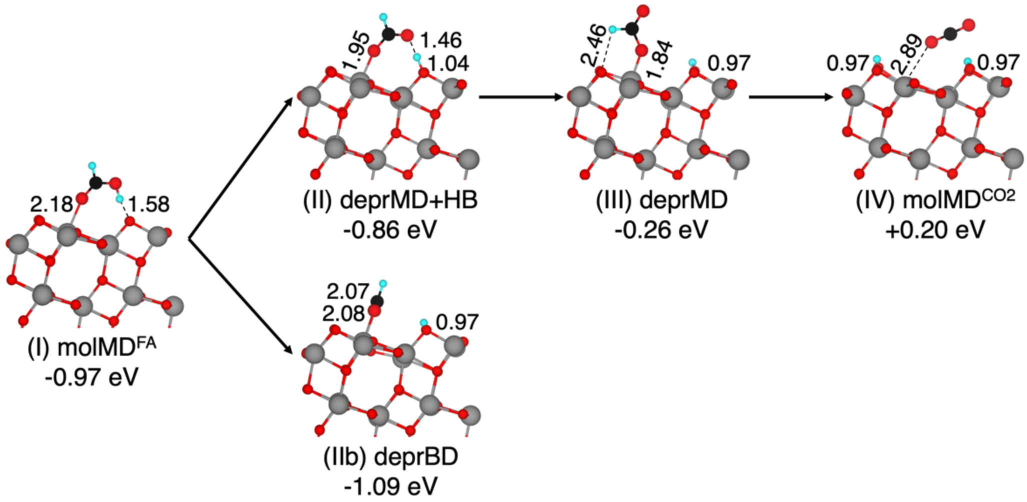

*Figure S2. Structures of formic acid thermal oxidation intermediates along the **inter**-pair route on the anatase TiO_2 (101) surface computed using the HSE06 density functional. Adsorption energies (in eV) and relevant bond lengths (in Å) are reported. White, black, red, and grey spheres represent H, C, O, and Ti atoms, respectively. For better visualization, FA spheres have been magnified.*

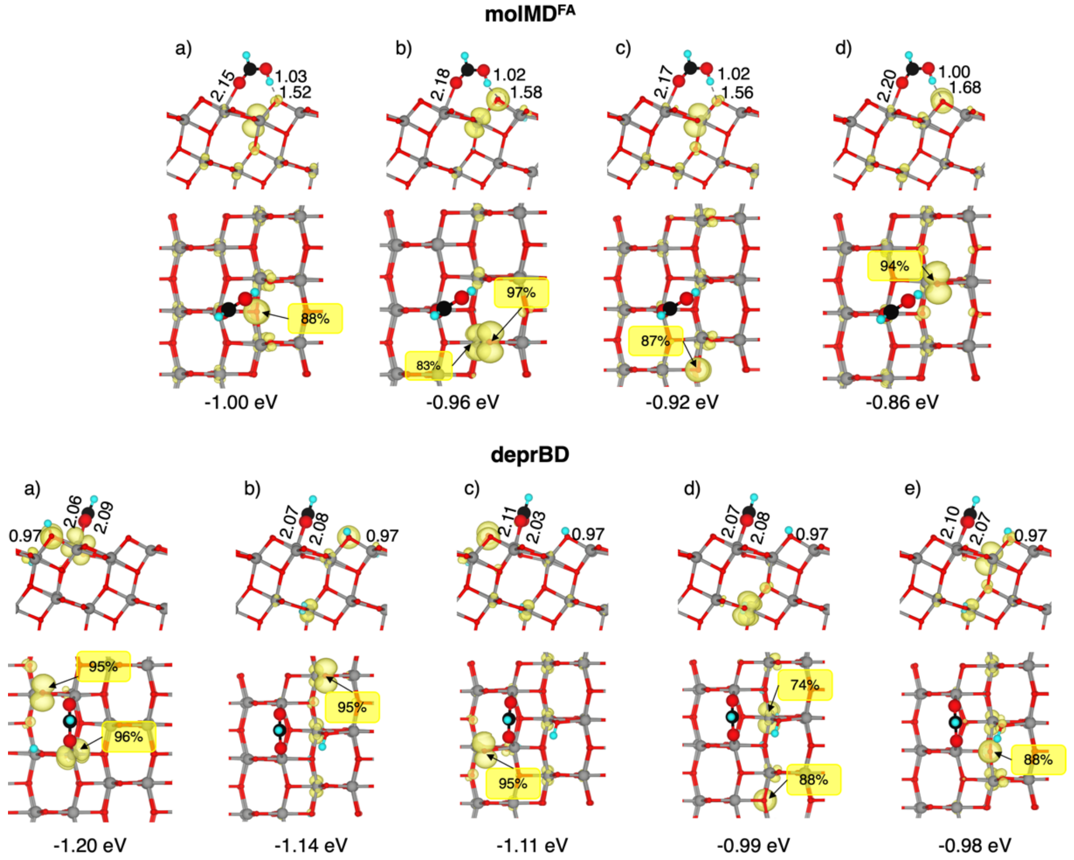

Figure S3. Structures of low-energy molecular monodentate ($molMD^{FA}$), and deprotonated bidentate (deprBD) configurations of adsorbed FA on the anatase TiO_2 (101) surface computed using the HSE06 density functional in the triplet photoexcited state. Adsorption energies (in eV) and selected bond lengths (in Å) are reported. White, black, red, and grey spheres represent H, C, O, and Ti atoms, respectively. Clouds of spin localization have been plotted in yellow on the adsorption structures with an isovalue of 0.005 a.u. using VESTA visualization software. For better visualization, FA spheres have been upscaled.

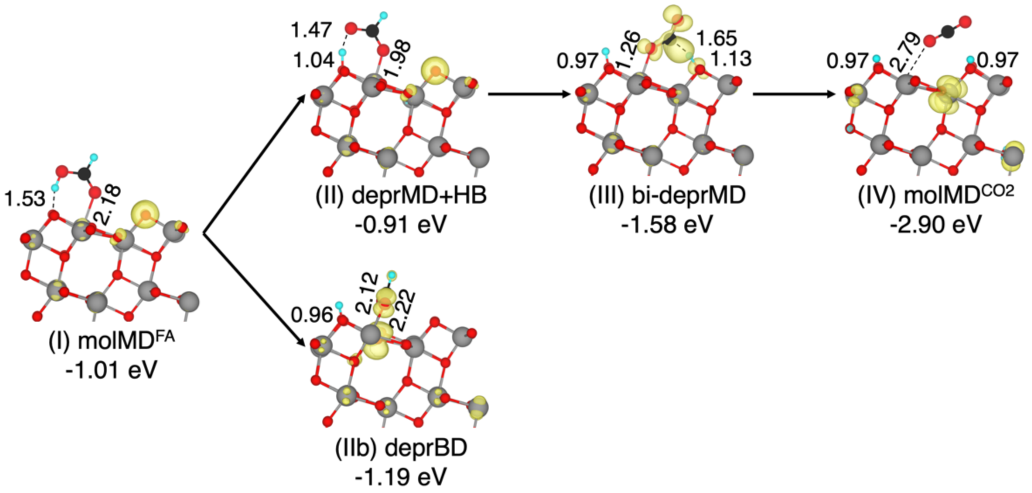

Figure S4. Structures of formic acid photocatalytic oxidation intermediates along the ***intra****-pair route on the anatase TiO_2 (101) surface computed using the HSE06 density functional. Adsorption energies (in eV) and relevant bond lengths (in Å) are reported. Cyan, black, red, and grey spheres represent H, C, O, and Ti atoms, respectively. Clouds of spin localization have been plotted in yellow with an isovalue of 0.005 a.u. using VESTA visualization software. For better visualization, FA spheres have been magnified.*

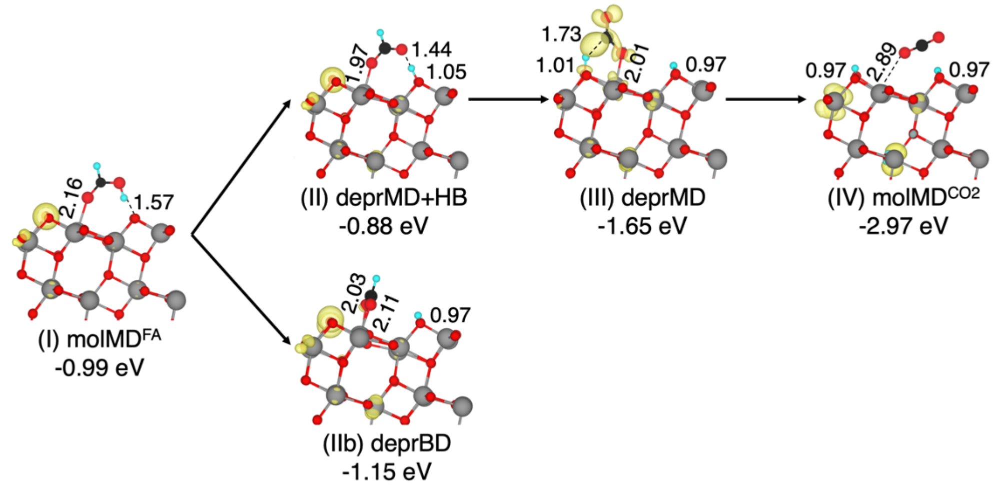

Figure S5. Structures of formic acid photocatalytic oxidation intermediates along the ***inter****-pair route on the anatase TiO_2 (101) surface computed using the HSE06 density functional. Adsorption energies (in eV) and relevant bond lengths (in Å) are reported. White, black, red, and grey spheres represent H, C, O, and Ti atoms, respectively. Clouds of spin localization have been plotted in yellow on the adsorption structures with an isovalue of 0.005 a.u. using VESTA visualization software. For better visualization, FA spheres have been magnified.*

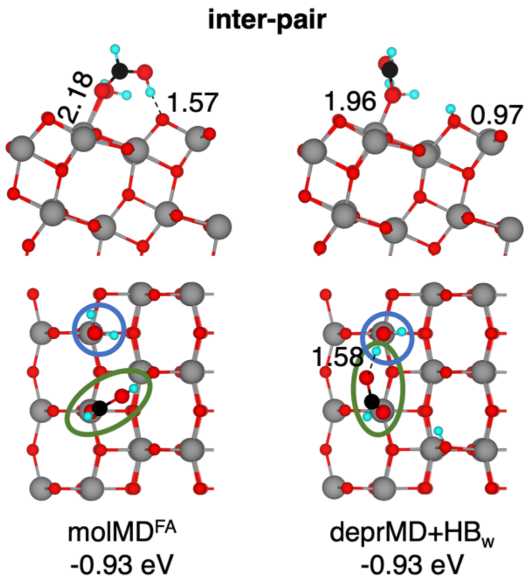

Figure S6. Alternative structures of molecular (molMD^{FA}) and deprotonated monodentate (deprMD+HB$_w$) inter-pair configurations of adsorbed FA on the anatase TiO_2 (101) surface in the presence of co-adsorbed water molecules computed using the HSE06 density functional in the singlet ground state. Adsorption energies (in eV) and selected bond lengths (in Å) are reported. White, black, red, and grey spheres represent H, C, O, and Ti atoms, respectively. On the top view, showing the top layer of TiO_2, blue and green circles highlight the position of adsorbed water and formic acid molecules on the surface. For better visualization, FA spheres have been magnified.

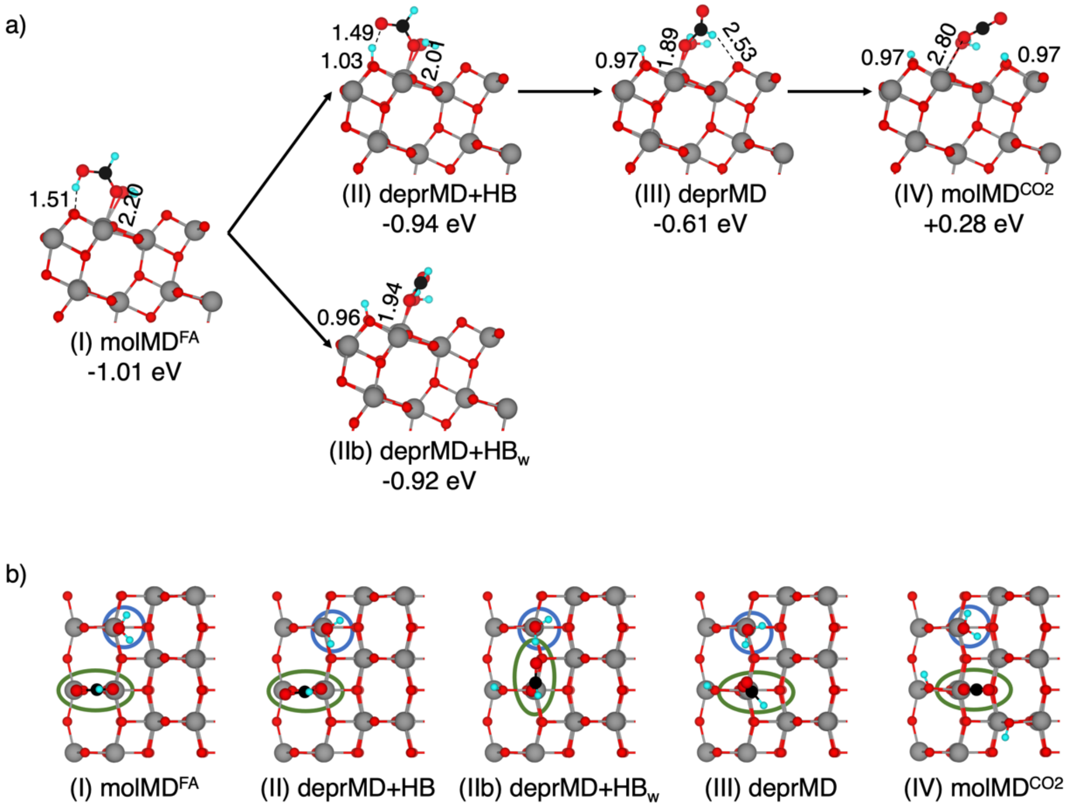

*Figure S7. Structures of formic acid thermal oxidation intermediates along the **intra**-pair route on the anatase TiO_2 (101) surface in the presence of co-adsorbed water molecules computed using the HSE06 density*

functional. On the side view (a) showing the first two layers of TiO_2, adsorption energies (in eV) and relevant bond lengths (in Å) are reported. On the top view (b) showing the top layer of TiO_2, blue and green circles highlight the position of adsorbed water and formic acid molecules on the surface. Cyan, black, red, and grey spheres represent H, C, O, and Ti atoms, respectively. For better visualization, FA spheres have been magnified.

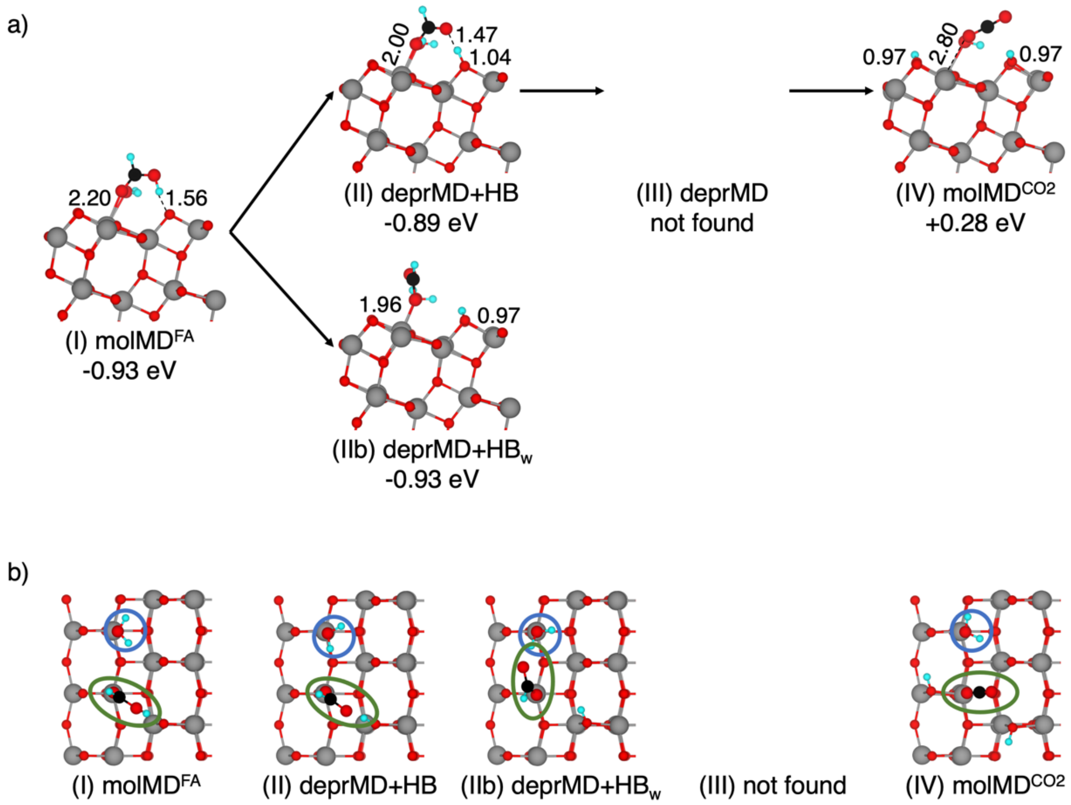

*Figure S8. Structures of formic acid thermal oxidation intermediates along the **inter**-pair route on the anatase TiO_2 (101) surface in the presence of co-adsorbed water molecules computed using the HSE06 density functional. On the side view (a) showing the first two layers of TiO_2, adsorption energies (in eV) and relevant bond lengths (in Å) are reported. On the top view (b) showing the first layer of TiO_2, blue and green circles highlight the position of adsorbed water and formic acid molecules on the surface. White, black, red, and grey spheres represent H, C, O, and Ti atoms, respectively. For better visualization, FA spheres have been magnified.*

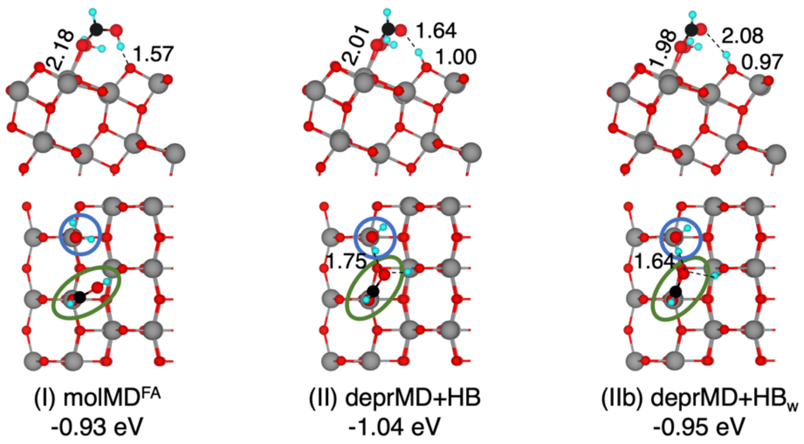

Figure S9. Alternative structures of formic acid thermal oxidation intermediates along the ***inter****-pair route on the anatase TiO_2 (101) surface in the presence of co-adsorbed water molecules computed using the HSE06 density functional. Adsorption energies (in eV) and relevant bond lengths (in Å) are reported. White, black, red, and grey spheres represent H, C, O, and Ti atoms, respectively. For better visualization, FA spheres have been magnified.*

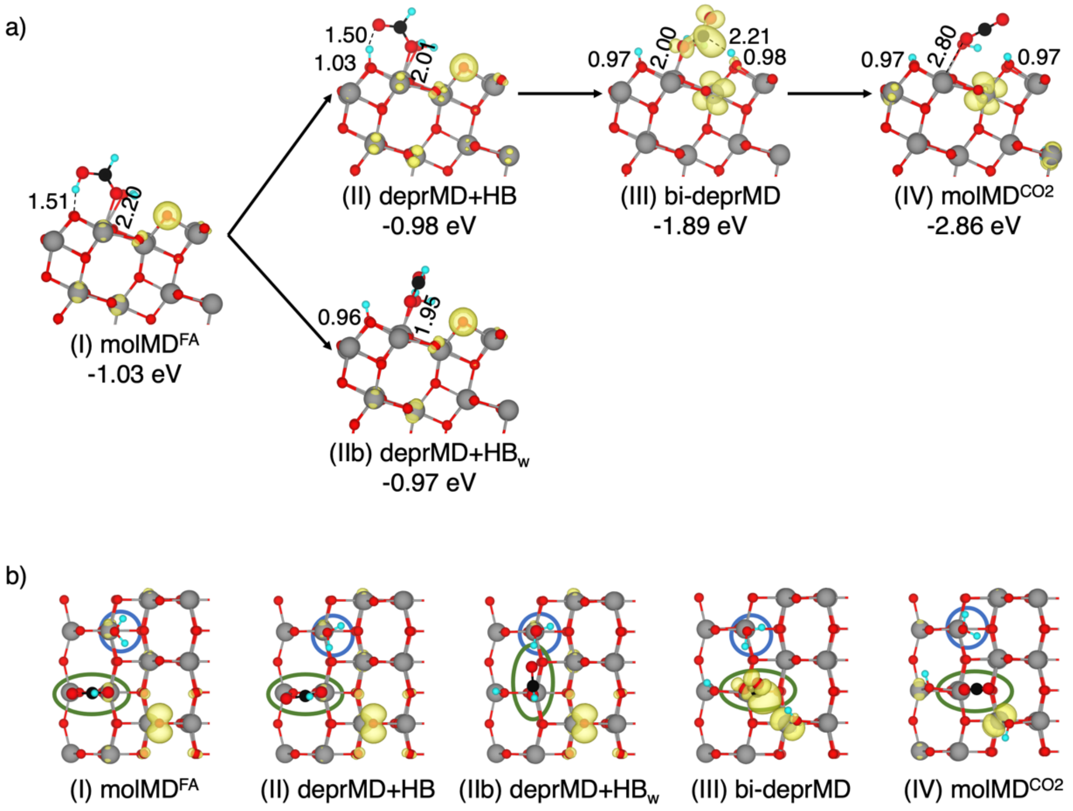

Figure S10. Structures of formic acid photocatalytic oxidation intermediates along the ***intra****-pair route on the anatase TiO_2 (101) surface in the presence of co-adsorbed water molecules computed using the HSE06 density functional. On the side view (a) showing the first two layers of TiO_2, adsorption energies (in eV) and relevant bond lengths (in Å) are reported. On the top view (b) showing the first layer of TiO_2, blue and green circles highlight the position of adsorbed water and formic acid molecules on the surface. Cyan, black, red, and grey spheres represent H, C, O, and Ti atoms, respectively. Clouds of spin localization have been plotted*

in yellow with an isovalue of 0.005 a.u. using VESTA visualization software. For better visualization, FA spheres have been magnified.

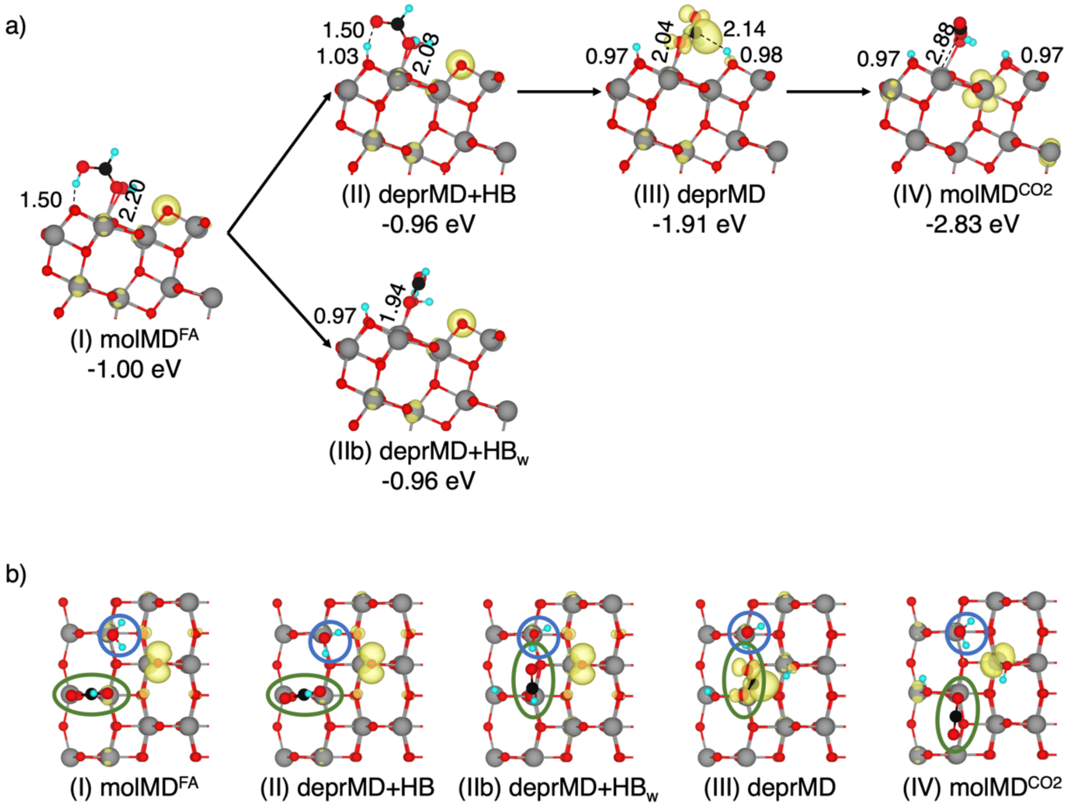

*Figure S11. Structures of formic acid photocatalytic oxidation intermediates along the **intra**-pair route on the anatase TiO_2 (101) surface in the presence of co-adsorbed water molecules computed using the HSE06 density functional. Compared to the reaction intermediates in S10 in the manuscript, the dissociated proton from the carboxylic group is transferred to a surface O_{2c} close to the adsorbed water molecule. On the side view (a) showing the first two layers of TiO_2, adsorption energies (in eV) and relevant bond lengths (in Å) are reported. On the top view (b) showing the first layer of TiO_2, blue and green circles highlight the position of adsorbed water and formic acid molecules on the surface. White, black, red, and grey spheres represent H, C, O, and Ti atoms, respectively. Clouds of spin localization have been plotted in yellow on the adsorption structures with an isovalue of 0.005 a.u. using VESTA visualization software. For better visualization, FA spheres have been magnified.*

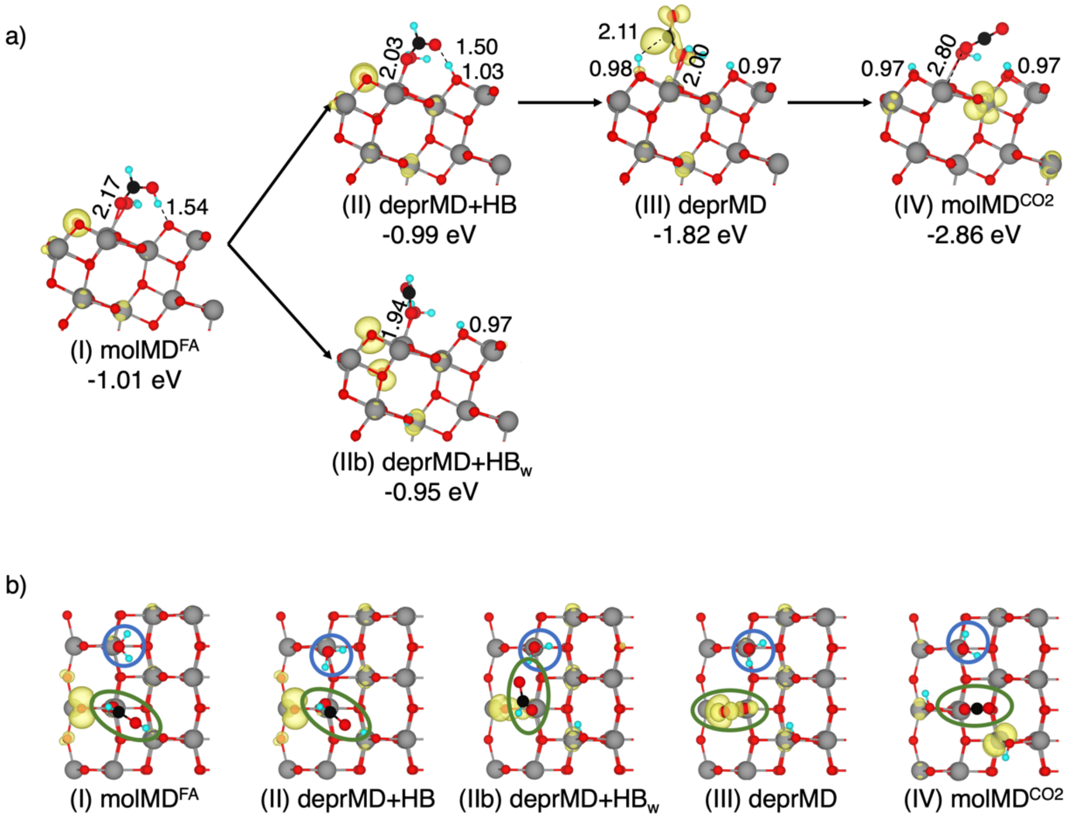

*Figure S12. Structures of formic acid photocatalytic oxidation intermediates along the **inter**-pair route on the anatase TiO_2 (101) surface in the presence of co-adsorbed water molecules computed using the HSE06 density functional. On the side view (a) showing the first two layers of TiO_2, adsorption energies (in eV) and relevant bond lengths (in Å) are reported. On the top view (b) showing the first layer of TiO_2, blue and green circles highlight the position of adsorbed water and formic acid molecules on the surface. White, black, red, and grey spheres represent H, C, O, and Ti atoms, respectively. Clouds of spin localization have been plotted in yellow on the adsorption structures with an isovalue of 0.005 a.u. using VESTA visualization software. For better visualization, FA spheres have been magnified.*

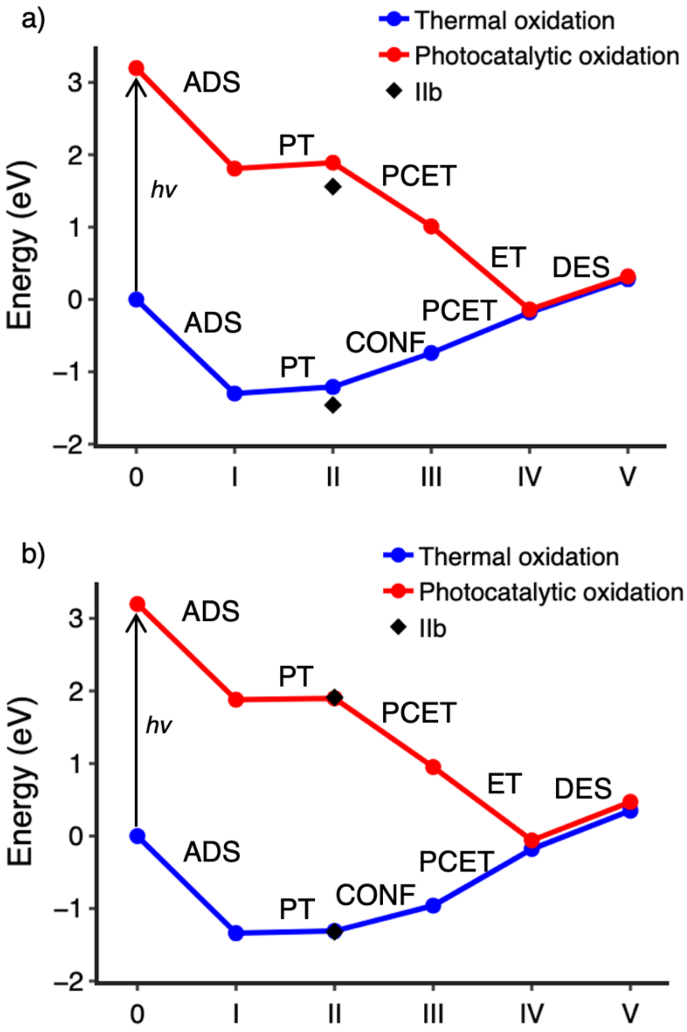

*Figure S13. Plots of the adsorption energy (as defined in Eq. 1) for the intermediates of the formic acid thermal (blue) and photocatalytic (red) oxidation reaction along the **intra**-pair route on anatase TiO_2 (101) in (a) anhydrous and (b) hydrated conditions, calculated with HSE06-D3. The excitation cost to start the photocatalytic reaction is highlighted by a black arrow. The zero-energy value is the sum of the energy of the reference anatase (101) slab, and the energy of isolated gas phase formic acid. Diamonds indicate the adsorption energy of the (IIb) intermediate (see text). Each step is labeled according to its nature: reactant adsorption (ADS), deprotonation (PT), proton-coupled electron transfer (PCET), geometry configuration rotation (CONF), and product desorption (DES).*

Table S1: Calculated adsorption energy values (obtained using the HSE06-D3 density functional and the definition in Eq. 1) for the intermediates of the formic acid thermal and photocatalytic oxidation reaction along all the intra-pair route on anatase TiO_2 (101) in anhydrous and hydrated conditions. For each system, the zero energy is the sum of the energy of the corresponding anatase (101) slab (as defined in Eq. 1 in the manuscript), and the energy of isolated gas phase formic acid.

**Intra-pair intermediates**	**E_{ads} (eV) anhydrous conditions**		**E_{ads} (eV) hydrated conditions**	
	**thermal**	**photocatalytic**	**thermal**	**photocatalytic**
0	0.00	0.00	0.00	0.00
I	-1.30	-1.39	-1.34	-1.32
II	-1.21	-1.31	-1.31	-1.30
IIb	-1.46	-1.64	-1.32	-1.29
III	-0.74	-2.19	-0.96	-2.25
IV	-0.18	-3.34	-0.18	-3.26
V	+0.28	-2.88	+0.35	-2.73

Table S2: Calculated adsorption energy values (obtained using the HSE06 density functional and the definition in Eq. 1) for the intermediates of the formic acid thermal and photocatalytic oxidation reaction along all the intra-pair and inter-pair routes on anatase TiO_2 (101) in anhydrous and hydrated conditions. For each system, the zero energy is the sum of the energy of the corresponding anatase (101) slab (as defined in Eq. 1 in the manuscript), and the energy of isolated gas phase formic acid.

**Intra-pair intermediates**	**E_{ads} (eV) anhydrous conditions**		**E_{ads} (eV) hydrated conditions**	
	**thermal**	**photocatalytic**	**thermal**	**photocatalytic**
**Figure ref.**	**Figure S1**	**Figure S4**	**Figure S7**	**Figure S10 (Figure S11)**
0	0.00	0.00	0.00	0.00
I	-0.99	-1.01	-1.01	-1.03 (-1.00)
II	-0.88	-0.91	-0.94	-0.98 (-0.96)
IIb	-1.03	-1.19	-0.92	-0.97 (-0.96)
III	-0.39	-1.58	-0.61	-1.89 (-1.91)
IV	+0.26	-2.90	+0.28	-2.86 (-2.83)
V	+0.38	-2.78	+0.51	-2.63
**Inter-pair intermediates**	**E_{ads} (eV) anhydrous conditions**		**E_{ads} (eV) hydrated conditions**	
	**thermal**	**photocatalytic**	**thermal**	**photocatalytic**
**Figure ref.**	**Figure S2**	**Figure S5**	**Figure S8 (Figure S9)**	**Figure S12**
0	0.00	0.00	0.00	0.00
I	-0.97	-0.99	-0.93 (-0.93)	-1.01
II	-0.86	-0.88	-0.89 (-1.04)	-0.99
IIb	-1.09	-1.15	-0.93 (-0.95)	-0.95
III	-0.26	-1.65	-	-1.82
IV	+0.20	-2.97	+0.28	-2.86
V	+0.38	-2.78	+0.51	-2.63

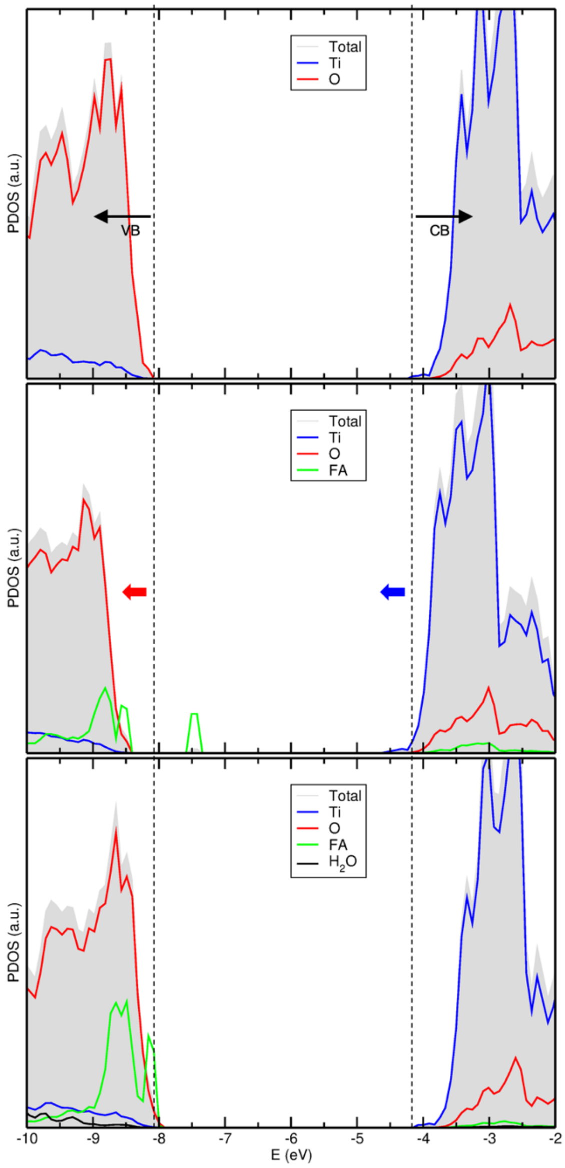

Figure S14. Projected Density of States (PDOS) for the anatase TiO_2 (101) surface slab bare (top panels), with one formic acid molecule adsorbed in the deprMD configuration without (central panels) and with co-adsorbed water molecules (bottom panels). The gray areas show the Total Density od States on top of which states of Ti and O atoms of the anatase slab, formic acid, and water atoms have been projected. Formic acid and water states have been rescaled (by 10) to make them more visible. The zero energy value of our plotted PDOS was set to vacuum.
